# A Cobalt Supramolecular Triple-Stranded Helicate-based Discrete Molecular Cage

**DOI:** 10.1038/srep43448

**Published:** 2017-03-06

**Authors:** Hien Duy Mai, Philjae Kang, Jin Kyung Kim, Hyojong Yoo

**Affiliations:** 1 Department of Chemistry, Hallym University, Chuncheon, Gangwon-do, 24252, Republic of Korea; 2 Department of Chemistry, Yonsei University, Seoul, 03722, Republic of Korea

## Abstract

We report a strategy to achieve a discrete cage molecule featuring a high level of structural hierarchy through a multiple-assembly process. A cobalt (Co) supramolecular triple-stranded helicate (Co-TSH)-based discrete molecular cage (**1**) is successfully synthesized and fully characterized. The solid-state structure of **1** shows that it is composed of six triple-stranded helicates interconnected by four linking cobalt species. This is an unusual example of a highly symmetric cage architecture resulting from the coordination-driven assembly of metallosupramolecular modules. The molecular cage **1** shows much higher CO_2_ uptake properties and selectivity compared with the separate supramolecular modules (Co-TSH, complex 2) and other molecular platforms.

Over the past decades, discrete metallosupramolecular platforms with a high degree of symmetry and intricacy have been extensively investigated owing to their promising applications in a variety of fields, including host-guest interactions^1^,^2^, catalysis^3^,^4^,^5^,^6^, drug delivery^7^,^8^, gas storage, and separation^9^,^10^,^11^. For example, molecular structures with large confined cavities created by the coordination-driven assembly of simple organic compounds and metals as the nodes have been reported^12^,^13^,^14^,^15^,^16^,^17^. The rational design of single molecular structures using multi-nuclear metal clusters (these were described as “secondary building blocks” in the previous reports)^18^,^19^,^20^,^21^,^22^,^23^ and various di- or multicarboxylate organic ligands has also been achieved^24^,^25^,^26^,^27^,^28^,^29^,^30^,^31^,^32^,^33^,^34^,^35^. Despite the structural distinction, their preparative paradigms are commonly confined by the use of simple coordination modes involving the assemblies of metal ions and ligands (“primary” assembly)^12^,^13^,^14^,^15^,^16^,^17^,^36^ or multinuclear metal clusters and multifunctional ligands (“secondary” assembly)^18^,^19^,^20^,^21^,^22^,^23^,^24^,^25^,^26^,^27^,^28^,^29^,^30^,^31^,^32^,^33^,^34^,^35^ (Fig. 1). Therefore, the diversity, symmetry, and complexity of targeted structures achieved by these conventional assembly methods are, to some extent, still limited.

A more advanced assembly mode can be used to produce discrete molecular platforms with a structural hierarchy that has previously been unattained in the field of metallosupramolecular chemistry. This assembly mode, corresponding to “tertiary” assembly, as shown in Fig. 1, is concerned with the use of predefined, well-organized, and secondarily-assembled metallosupramolecular modules, permitting access to systematic molecular architectures. In other words, such molecules can be built from three distinct levels of structural hierarchy. However, it should be emphasized that tertiary assemblies are discrete rather than infinite, highlighting their difference from polymeric frameworks^18^,^19^,^20^,^21^,^22^,^23^,^37^. Clear-cut innovations of tertiary assembly over the corresponding primary and secondary assemblies can be addressed as follows: first, structurally well-organized, highly-ordered metallosupramolecular modules acting as the basic platforms can provide diverse assembly fashions. This enables the construction of hierarchical discrete platforms possessing target-oriented properties that can rarely be obtained in individual units. Second, the resultant molecules would have the maximum expression of unique functional sites in specific geometrical arrangements through rational supramolecular module design and appropriate self-assembly considerations.

We report a discrete molecular platform with a high level of structural hierarchy through the coordination-driven assembly of supramolecular modules. A cobalt supramolecular triple-stranded helicate (Co-TSH)-based discrete high-order molecular cage is successfully synthesized. PDA ligand is chosen due to its considerable importance in the formation of tetranuclear cobalt cluster as a primary assembly (PDA = 2,6-pyridinedicarboxylate)^35^. Two tetranuclear clusters with *tbu*-PTA generate a Co-TSH as a conceptual secondary assembly (*tbu*-PTA = 5-tert-butyl isophthalate). The six well-organized Co-TSHs generated *in-situ* are supramolecular modules; their assembly leads to the formation of a discrete molecular cage. Moreover, the synthesized molecular cage shows a significant enhancement in selective CO_2_ capture over other gases under ambient conditions compared with other single molecules constructed from metal ions/clusters and organic ligands.

## Results and Discussion

Treatment of 3 equiv. of Co(CH_3_COO)_2_·4H_2_O, 2 equiv. of 2,6-pyridinedicarboxylic acid (H_2_PDA), and 1 equiv. of 5-tert-butyl isophthalic acid (H_2_*tbu*-PTA) in DMF (dimethylformamide) at 100 °C for 6 h leads to the formation of {[Co_8_(PDA)_6_(*tbu*-PTA)_3_(DMF)_4_(H_2_O)_2_]_6_-[Co(H_2_O)_3_]_4_} (**1**) (Fig. 2).

Complex **1** is isolated as purple rectangular crystals (42.35% yield based on the amount of H_2_PDA used). The solid-state structure of **1** is determined by single-crystal X-ray diffraction (SXRD) analysis, and solved and refined as the space group of *Fd-3* (Fig. 3 and Supplementary Table S1). The solid-state structure of **1** shows that it is composed of six Co-TSHs interconnected by the four linking cobalt atoms (Co5) (Fig. 3A–C and Supplementary Fig. S1). Each Co-TSH, comprising two tetranuclear Co clusters linked together by three *tbu*-PTA ligands (Supplementary Fig. S2), is geometrically similar to that reported previously^35^. Each Co-TSH forms coordination bonds with two Co5 atoms through its unoccupied carboxylate oxygen atoms (Supplementary Fig. S3), and each Co5 atom with a pseudo-octahedral coordination geometry is interconnected to three neighboring Co-TSHs (Supplementary Fig. S4). It should be emphasized that the generation of **1** is strongly governed by two important factors: (a) the availability, position, and orientation of unoccupied oxygen donors on Co-TSHs and (b) the coordination environment of the Co5 atoms. In (a), considering the *tbu*-PTA ligands on each Co-TSH as the “strands”, the two oxygen atoms (two O12 atoms from the same Co-TSH) that are directly bound to different Co5 atoms lie almost opposite to each other. Those oxygen atoms are on the peripheral points of extension of the strand *tbu*-PTA with a tert-butyl group orientating towards the center (Supplementary Fig. S3). This coordination mode of Co-TSH to the Co5 atom could minimize the steric hindrance of other neighboring Co-TSHs. Regarding factor (b), each six-coordinate Co5 atom bonds to the three *tbu*-PDA ligands, each belonging to three neighboring Co-TSHs, in a facial (*fac*−) mode (inset in Fig. 3A and Supplementary Fig. S4B). This *fac*-coordination mode, coupled with a suitable orientation of the interaction sites on Co-TSHs, allows the coordinative assembly of six Co-TSHs into discrete molecular cage.

The longest transverse distance of **1** is ca. 39 Å. The molecular cage **1** contains a confined space that could be viewed as a pseudo-regular tetrahedron (Fig. 3D and Supplementary Fig. S5), of which each vertex is occupied by a Co5 atom with an edge distance (Co5∙∙∙Co5 separation) of ca. 17.3 Å. The measurement of temperature-dependent magnetization (emu/g) of **1** has been carried out using a Quantum Design MPMS-5XL magnetometer for temperatures 4 K ≤ *T* ≤ 300 K with a 500 Oe applied fields (Supplementary Fig. S6A). The magnetic behavior of **1** is described by Curie-Weiss law^38^,^39^,^40^,^41^; and the corresponding fitting (1/*χ* vs *T*) (Supplementary Fig. S6B) yields a value of *θ* = −9.92 K from the intercept, which suggests the antiferromagnetic interaction between cobalt ions^40^,^42^,^43^, and *C* =  0.0103 emu K g^−1^ from the slope (1/*C*). The measurement *χ*_*M*_*T* at 300 K is ca. 2.86 (emu K mol^−1^) (Supplementary Fig. S6C). This value, while higher than the estimated spin-only value of 1.88 (emu K mol^−1^) for *S* = 3/2, still falls within an acceptable range when compared to other experimentally observed high-spin octahedral Co (II) ions with an orbital angular momentum contribution^42^,^43^,^44^,^45^,^46^. Upon cooling, *χ*_*M*_*T* continuously decreases to a value of 1.73 (emu K mol^−1^) at 6.4 K. The chemical states of the Co species in **1** are also investigated by X-ray photoelectron spectroscopy (XPS) (Supplementary Fig. S7). An intense and characteristic satellite at *ca*. 786 eV (no other satellites appearing in the area of over 790 eV from the Co 2p_1/2_ and Co2p_3/2_ spectra) indicates that all cobalts show 2+ states^47^. This result is further confirmed by calculating the bond valence sums using the observed bond distances in the crystal structure data (Supplementary Table S3)^48^,^49^. The phase purity of the as-synthesized **1** is confirmed using powder X-ray diffraction (PXRD) (Supplementary Fig. S8). The data show considerable similarities between the experimental and simulated PXRD patterns. The crystalline stability is proved to be retained below 120 °C by variable temperature PXRD experiments (Supplementary Figs S9 and S10). The thermogravimetric analysis (TGA) (Supplementary Fig. S11) of **1** shows minor weight loss below 350 °C, which is attributed to the removal of coordinated DMF and H_2_O molecules. Above 350 °C, the complete decomposition of **1** occurred.

**Figure 1:**
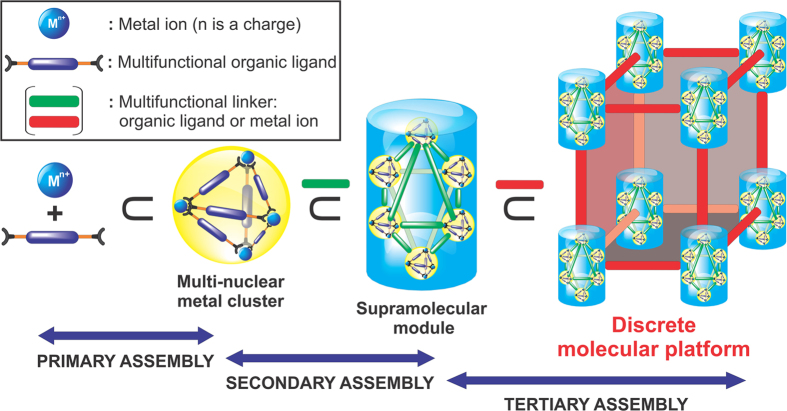
Conceptual representation of the general coordination-driven assembly modes of molecular architectures in terms of increasing levels of structural hierarchy and complexity. Note that multifunctional organic ligands or metal ions can be regarded as multifunctional in secondary and tertiary assemblies.

**Figure 2:**
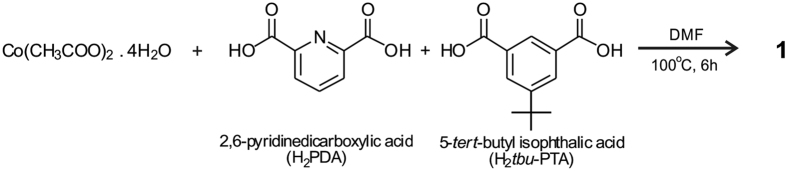
Reaction for the molecular cage **1**.

**Figure 3:**
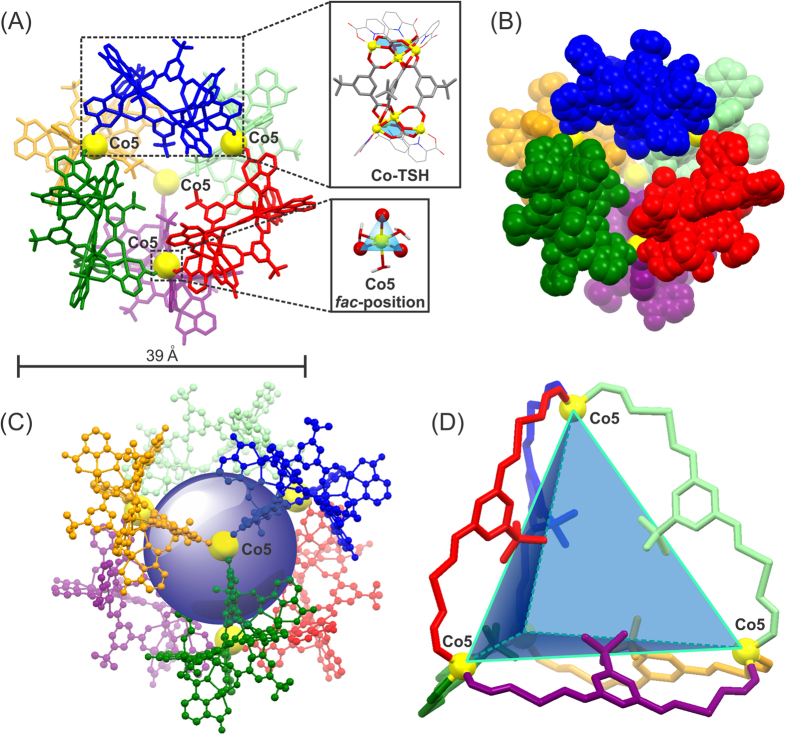
X-ray crystal structure of discrete molecular cage **1**. (**A**) **1** is composed of six Co-TSHs (portrayed in different colors) interconnected by four Co5 atoms (yellow balls). Insets in (**A**) (top) crystal structure of each Co-TSH generated *in situ* and (bottom) coordination mode of each Co5 atom. The corresponding (**B**) space-filling and (**C**) ball-and-stick representations of **1**. (**D**) Simplified structure of **1**, in which only the extended strands from Co-TSHs that directly connect to Co5 atoms are shown, highlighting a large regular tetrahedron of **1** defined by four Co5 atoms. The blue sphere in (**C**) delineates the confined space at the center of the molecular cage **1**. In (**A**) to (**C**), all the coordinated and free solvents, hydrogen atoms, and disorder components of *tert*-butyl groups are omitted for clarity. Color of atoms for insets in (**A**) C, grey; N, blue; O, red; Co, yellow.

It is interesting to note that the molecular cage **1** is considered a result of the assembly of Co-TSHs, as shown in Fig. 1. Although similar concepts of higher-order discrete platforms based on supramolecular assemblies have been previously demonstrated^50^,^51^, they are mainly based on noncoordinative interactions (e.g. van der Waals, electrostatic, π–π interaction, or hydrogen bonding). Notably, all the assemblies that form **1** are coordination-driven. It is well established that coordination-driven motifs not only affect the stability, but also often provide unique design features in the assemblies because of the higher directionality offered by metal–ligand coordinative bonding^36^. To the best of our knowledge, and based on our conceptual assembly model (Fig. 1), the molecular cage **1** is the first example of a discrete cage architecture exhibiting an unprecedentedly higher order of hierarchy resulting from the metal-directed tertiary assembly of preassembled secondary metallosupramolecules.

The discrete cobalt triple-stranded helicate (Co-TSHs) is successfully prepared by separate experiments. Treatment of 2 equiv. of Co(NO_3_)_2_·6H_2_O, 1 equiv. of H_2_PDA, and 1 equiv. of H_2_*tbu*-PTA in DMF at 120 °C for 36 h affords {Co_8_(PDA)_6_(*tbu*-PTA)_3_(DMF)_6_} (**2**). Complex **2** is isolated as purple rhombic crystals. The solid-state structure of **2** is determined by SXRD, and solved and refined as the space group of *P2*_*1*_/*n* (Supplementary Fig. S13 and Supplementary Table S2). The solid-state structure of **2** shows that two distinct conformations, left- and right-handed, exist simultaneously in a single unit cell. Apart from the orientation difference of interconnecting *tbu*-PTA ligands, both conformations are geometrically similar to each other (Supplementary Fig. S13). It should be noted that the left-handed conformation is topologically similar to that reported previously^35^, whereas the right-handed conformation shows a structure similar to that of a basic supramolecular module of **1**. Variable-temperature (4–300 K) magnetic measurement of complex **2** is conducted under an applied field of 500 Oe (Supplementary Fig. S14A). Curie-Weiss fitting (1/*χ* versus *T*) yields a value of *θ* = −23.85 K and *C* = 0.0086 emu K g^−1^ (Supplementary Fig. S14B) and the value *χ*_*M*_*T* at 300 K is found to be 2.59 (emu K mol^−1^) which could be assigned to high-spin octahedral Co(II) ions (Supplementary Fig. S14C)^42^,^43^,^44^,^45^,^46^. The existence of Co(II) in complex **2** is also confirmed by XPS (Supplementary Fig. S15) and calculation of the bond valence sums using the observed bond distances in the crystal structure data (Supplementary Table S4). The phase purity of **2** is confirmed by a good match between the experimental and simulated PXRD patterns (Supplementary Fig. S16). The TGA (Supplementary Fig. S17) also indicates that the removal of coordinated DMF and H_2_O in **2** occurred below 350 °C.

The transformation of **2** to **1** is confirmed by XRD experiments. In the treatment of **2** with excess Co(NO_3_)_2_·6H_2_O in DMF at 50 °C, slow generation of rectangular crystals is observed (**2** generally shows rhombic crystals, Supplementary Fig. S19). The SXRD and PXRD analyses confirm that the newly-generated crystals in the reaction mixture are **1** (Supplementary Fig. S20). The assembly processes to give the discrete molecular cage can be achieved by both direct and step-wise approach.

**Figure 4:**
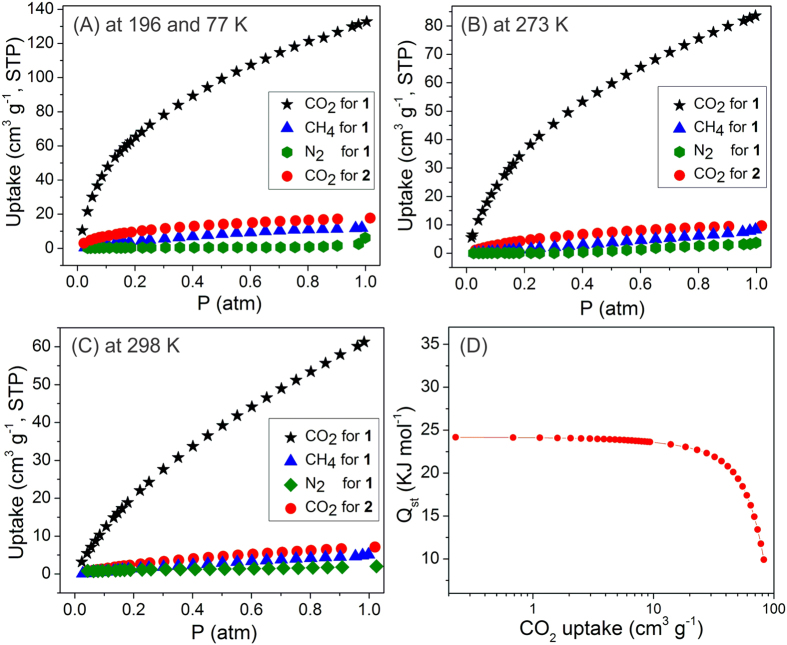
Adsorption isotherms of discrete molecular cage 1 for CO_2_, CH_4_, and N_2_ and that of complex 2 for CO_2_ collected at different temperatures. (**A**) at 196 K (for CO_2_ and CH_4_) and 77 K (for N_2_), (**B**) at 273 K, and (**C**) at 298 K. (**D**) Isosteric heat of adsorption of **1**.

Efficient CO_2_ capture and separation play a vital role in both environmental and industrial applications. In particular, a high selectivity of CO_2_ over other components of gas mixtures is essential^52^,^53^,^54^. The CO_2_ capture performances and selectivity over other gases are tested (e.g. N_2_ and CH_4_) at room temperature and ambient pressure. Prior to the gas adsorption experiments, the solvent molecules of **1** are removed by successive acetone solvent exchanges and heating at 60 °C under a vacuum for 24 h. After the activation step, the crystallinity of **1** is still maintained, as confirmed by the PXRD analysis (Supplementary Fig. S8). The gas adsorption measurements at 196 K (for CO_2_ and CH_4_) and 77 K (for N_2_) (Fig. 4A) show significant uptakes of CO_2_ (ca. 132 cm^3^ g^−1^) for **1**, almost excluding N_2_ and CH_4_ (ca. 6.1 and 11.9 cm^3^ g^−1^, respectively). Moreover, as shown in Fig. 4B and C, **1** has a high CO_2_ uptake (83 and 61 cm^3^ g^−1^), but slight CH_4_ (8.3 and 5.1 cm^3^ g^−1^) and N_2_ (3.7 and 2.0 cm^3^ g^−1^) adsorption capacities at 273 and 298 K, respectively (at 1 atm). High selective CO_2_ adsorption over other gases can be attributed to Co sites, particularly Co5, resulting from the desolvation step, which could induce better interactions with a higher quadrupole moment and polarizability of CO_2_ compared with CH_4_ and N_2_^52^,^55^,^56^,^57^. The CO_2_ isosteric heat of adsorption (Q_st_) of **1** (Fig. 4D), calculated by fitting the 273 and 298 K isotherms to the virial-type expression^41^, is found to be ca. 24.1 kJ mol^−1^ at a low loading. This Q_st_ value falls within the range of most materials with high CO_2_ adsorption capacity caused by exposed metal sites^52^. A variety of materials with high affinity towards CO_2_ caused by favored interactions with exposed metal sites have also been reported^52^,^58^,^59^. Given that CO_2_ molecule has a lower kinetic diameter than those of CH_4_ and N_2_^52^,^55^,^56^,^57^, the confined cage structure of **1** could preferentially entrap CO_2_, thus improving the CO_2_ selectivity over others. The CO_2_ adsorptions of **2** exhibit much lower uptakes of 17.7, 9.7, and 7.1 cm^3 ^g^−1^ at 196, 273, and 298 K at 1 atm, respectively (Fig. 4A–C). This remarkable difference in adsorption suggests that confined cavities of **1** significantly enhance the CO_2_ adsorption performance of **1**. Complex **2** also shows little adsorption towards CH_4_ and N_2_ (Supplementary Figs S21–S23). To the best of our knowledge, the CO_2_ uptake capacity of **1** at 298 K and 1 atm is among the highest values for discrete molecular platforms constructed from metal ions/clusters with organic ligands (Supplementary Table S5)^9^,^10^,^11^,^60^,^61^,^62^,^63^,^64^,^65^,^66^,^67^,^68^.

## Conclusions

In conclusion, a facile strategy is developed to achieve a discrete molecular platform through the assembly of well-organized supramolecules. A novel cobalt supramolecular triple-stranded helicate (Co-TSH)-based molecular platform, (**1**), is successfully synthesized. Complex **1** is a structurally well-defined, highly systematic, and discrete cage architecture resulting from the coordination-driven assembly of *in-situ*-generated supramolecular modules. The right- and left-handed Co-TSHs, (**2**), structurally analogous to the Co-TSHs of **1**, are also successfully isolated from a separate reaction. The molecular cage **1** shows much higher CO_2_ capture capacity and selectivity compared with **2** and other single molecules, including cage complexes. The CO_2_ uptake capacity of **1** at 298 K and 1 atm is among the highest values for discrete molecular platforms constructed from metal ions/clusters.

## Methods

### Synthesis of {[Co_8_(PDA)_6_(*tbu*-PTA)_3_(DMF)_4_(H_2_O)_2_]_6_-[Co(H_2_O)_3_]_4_} (1)

To a mixed-ligand DMF solution (9 mL) of H_2_PDA (50.14 mg, 0.30 mmol) and H_2_*tbu*-PTA (33.34 mg, 0.15 mmol) was added a DMF solution (9 mL) of Co(OAc)_2_ · 4H_2_O (112.09 mg, 0.45 mmol) in a 20 mL glass vial at room temperature. The vial was sealed tightly, and heated to 100 °C (increasing rate; 2.67 °C/min), and maintained at this temperature for 6 h. Afterward, the mixture was gradually cooled to 30 °C with a cooling rate of −0.25 °C/min. Purple rectangular crystals were collected, washed sequentially with DMF (3 × 10 mL) and acetone (3 × 10 mL), and dried under vacuum at room temperature.

### Synthesis of {Co_8_(PDA)_6_(*tbu*-PTA)_3_(DMF)_6_} (2)

Co(NO_3_)_2_ · 6H_2_O (87.31 mg, 0.3 mmol), H_2_PDA (25.07 mg, 0.15 mmol), H_2_*tbu*-PTA (33.34 mg, 0.15 mmol), HCl (0.5 mL, 0.05 mmol), and DMF (12.5 mL, 161.44 mmol) were mixed in a 20 mL vial at room temperature. The vial was sealed tightly and heated to 120 °C (increasing rate; 3 °C/min). Then the reaction mixture was maintained at 120 °C for 36 h, and cooled to 30 °C (cooling rate; −0.05 °C/min). Purple rhombic crystals were collected, washed sequentially with DMF (3 × 10 mL) and acetone (3 × 10 mL) and dried under vacuum.

### Transformation of 2 to 1

Complex **2** (38.96 mg, 0.015 mmol), Co(NO_3_)_2_ · 6H_2_O (34.92 mg, 0.12 mmol), and DMF (6 mL) are mixed in a 20 mL vial at room temperature. The vial is sealed tightly and heated to 50 °C, and then the reaction mixture is maintained for 3 days, and cooled down to room temperature. The generation of purple rectangular crystals begins to be observable after 4 hours, and more formed as the reaction proceeded. Purple rectangular crystals are collected and analyzed through the single crystal X-ray diffraction (SXRD) and powder X-ray diffraction (PXRD) methods.

### Single crystal X-ray diffraction

The diffraction data were collected at 100 K on a ADSC Quantum 210 CCD diffractometer equipped with synchrotron radiation (0.75000 Å) at the Supramolecular Crystallography 2D, Pohang Accelerator Laboratory (PAL), Pohang, Korea. Crystal structures were solved using the direct method with SHELX-XT (Ver. 2014/5) and refined by full-matrix least-squares calculations with the SHELX-XL (Ver. 2014/7) program package. Detailed descriptions of SXRD analysis are given in the Supplementary Information.

Crystallographic data have been deposited with the Cambridge Crystallographic Data Centre: CCDC 1449883 (complex **1**) and CCDC 1449884 (Complex **2**).

### Gas adsorption measurements

Gas adsorption isotherms were obtained using BELSORP-mini II (BEL Japan, Inc.). The gases used throughout adsorption experiments were highly pure (99.999%). Prior to the adsorption experiments, all the samples were activated as follows: First, the as-synthesized sample was thoroughly rinsed with DMF (3 × 10 mL) and immersed in 10 mL acetone for 24 h for solvent exchange; the acetone was decanted and replenished with fresh solvent. This procedure was repeated three times. Finally, the sample was dried under vacuum at 60 °C for 24 h prior to the gas sorption measurements.

## Additional Information

**How to cite this article**: Mai, H. D. *et al*. A Cobalt Supramolecular Triple-Stranded Helicate-based Discrete Molecular Cage. *Sci. Rep.* **7**, 43448; doi: 10.1038/srep43448 (2017).

**Publisher's note:** Springer Nature remains neutral with regard to jurisdictional claims in published maps and institutional affiliations.

## Supplementary Material

Supporting Information
