# Supplementary material for: A Cobalt Supramolecular Triple-Stranded Helicate-based Discrete Molecular Cage
Source: Sci Rep. 2017 Mar 6;7:43448. doi: 10.1038/srep43448 (PMC5337952; doi:10.1038/srep43448)
Supplement: Supporting Information [file srep43448-s1.pdf]

Supplementary Information for

# A Cobalt Supramolecular Triple-Stranded Helicate-based Discrete Molecular Cage

Hien Duy Mai,[a] Philjae Kang,[b] Jin Kyung Kim,[a] and Hyojong Yoo*[a]

[a]Department of Chemistry, Hallym University, Chuncheon, Gangwon-do, 24252, Republic of Korea

[b]Department of Chemistry, Yonsei University, Seoul, 03722, Republic of Korea

Correspondence to: hyojong@hallym.ac.kr

**This PDF file includes:**

Materials and Methods
Figures S1 to S23
Tables S1 to S5

**Materials and Methods**

**General Methods**

All the glassware is oven-dried prior to use. The IR spectra of the complexes are recorded in the 399–4000 $cm^{-1}$ range using KBr pellets on a Nicolet iS5 FT-IR (ThermoScientific) spectrometer. Thermogravimetric analysis (TGA) is performed using a TA Instruments SDT Q600 analyzer under a nitrogen atmosphere from 18 to 600 °C at a heating rate of 2 °C/min. X-ray photoelectronic spectroscopy (XPS) measurement is performed on a K-ALPHA spectrometer (Thermo VG, United Kingdom) with monochromated Al Kα X-ray radiation as the X-ray source for excitation. Magnetic measurements of compounds are performed using a Quantum Design MPMS-5XL magnetometer for temperatures $4 K \leq T \leq 300 K$ with a 500 Oe applied fields. Powder X-ray diffraction (PXRD) analysis is performed on a RIGAKU Ultima IV diffractometer using Cu Kα radiation (wavelength 1.541 Å) in the focused beam configuration with a continuous scan rate of 2° $min^{-1}$ in the 3–50° range at room temperature. Simulated PXRD patterns are calculated from single crystal X-ray diffraction (XRD) data using the *Mercury 3.3* program. Elemental analyses are performed on a Thermo Finnigan Flash EA1112 unit. Gas adsorption isotherms are obtained using BELSORP-mini II (BEL Japan, Inc.). The gases used throughout adsorption experiments are highly pure (99.999%). Prior to the adsorption experiments, all the samples are activated as follows: First, the as-synthesized sample is thoroughly rinsed with DMF (3 × 10 mL) and immersed in 10 mL acetone for 24 h for solvent exchange; the acetone is decanted and replenished with fresh solvent. This procedure is repeated three times. Finally, the sample is dried under vacuum at 60 °C for 24 h prior to the gas sorption measurements. Adsorption experiments are carried out at four different adsorption temperatures, 77, 196, 273, and 298 K, attained using liquid nitrogen, a

mixture of dry ice and 2-propanol, a mixture of ice and water, and water, respectively. Optical microscope images are acquired with Nikon Eclipse 80*i* microscope and Canon EOS 80D camera.

**Materials**

Cobalt(II) nitrate hexahydrate ($Co(NO_3)_2{\cdot}6H_2O$, 98 %, Sigma-Aldrich), cobalt(II) acetate tetrahydrate ($Co(OAc)_2{\cdot}4H_2O$, ≥ 98 %, Sigma-Aldrich), 2,6-pyridinedicarboxylic acid ($C_7H_5NO_4$, 99%, Sigma-Aldrich), 5-*tert*-butyl isophthalic acid ($C_{12}H_{14}O_4$, 99%, Sigma-Aldrich), N,N-dimethylformamide (DMF, 99.99%, Burdick & Jackson), and HCl (extra pure, Burdick & Jackson) are used as received. Abbreviations used: OAc = acetate, PDA = 2,6-pyridinedicarboxylate, $H_2$PDA = 2,6-pyridinedicarboxylic acid, *tbu*-PTA = 5-*tert*-butyl isophthalate, $H_2$*tbu*-PTA = 5-*tert*-butyl isophthalic acid.

**Synthesis of the molecular cage, {[$Co_8$(PDA)$_6$(*tbu*-PTA)$_3$(DMF)$_4$($H_2O$)$_2$]$_6$-[Co($H_2O$)$_3$]$_4$]} (1)**

To a mixed-ligand DMF solution (9 mL) of $H_2$PDA (50.14 mg, 0.30 mmol) and $H_2$*tbu*-PTA (33.34 mg, 0.15 mmol) is added a DMF solution (9 mL) of $Co(OAc)_2 \cdot 4H_2O$ (112.09 mg, 0.45 mmol) in a 20 mL glass vial at room temperature. The vial is sealed tightly, and heated to 100 °C (increasing rate; 2.67 °C/min), and maintained at this temperature for 6 h. Afterward, the mixture is gradually cooled to 30 °C with a cooling rate of –0.25 °C/min. Purple rectangular crystals are collected, washed sequentially with DMF (3 × 10 mL) and acetone (3 × 10 mL), and dried under vacuum at room temperature. Yield: 53.5 mg, 42.35 % (based on $H_2$PDA). Anal. Calcd. for $C_{540}H_{540}Co_{52}N_{60}O_{264}$ = [[$Co_8$(PDA)$_6$(*tbu*-PTA)$_3$(DMF)$_4$($H_2O$)$_2$]$_6$-[Co($H_2O$)$_3$]$_4$] (FW = 15158.81 g/mol): C, 42.79; H, 3.59; N, 5.54. Found: C, 42.41; H, 3.52; N, 4.92. FT-IR (KBr, 4000 ~ 500 $cm^{-1}$): the band at *ca.* 3432 $cm^{-1}$ attributed to ν(OH); the band at *ca.* 2970 $cm^{-1}$ corresponds to ν(C-

H); the bands at *ca.* 1623 and 1573 $cm^{-1}$ correspond to asymmetric stretching of carboxylates groups whereas those at *ca.* 1435 and 1383 $cm^{-1}$ correspond to symmetric stretching of the carboxylates groups (Supplementary Fig. S12).[1]

**Synthesis of {$Co_8(PDA)_6$(*tbu*-PTA)$_3$(DMF)$_6$} (2)**

$Co(NO_3)_2 \cdot 6H_2O$ (87.31 mg, 0.3 mmol), $H_2PDA$ (25.07 mg, 0.15 mmol), $H_2$*tbu*-PTA (33.34 mg, 0.15 mmol), HCl (0.5 mL, 0.05 mmol), and DMF (12.5 mL, 161.44 mmol) are mixed in a 20 mL vial at room temperature. The vial is sealed tightly and heated to 120°C (increasing rate; 3 °C/min). Then the reaction mixture is maintained at 120 °C for 36 h, and cooled to 30 °C (cooling rate; −0.05 °C/min). Purple rhombic crystals are collected, washed sequentially with DMF (3 × 10 mL) and acetone (3 × 10 mL) and dried under vacuum. Yield: 27.8 mg, 43.50 % (based on $H_2PDA$). Anal. Calcd. for $C_{96}H_{96}Co_8N_{12}O_{42}$ = $Co_8(PDA)_6$(*tbu*-PTA)$_3$(DMF)$_6$: C, 45.02; H, 3.78; N, 6.56. Found: C, 44.17; H, 3.99; N, 5.92. FT-IR (KBr, 4000 ~ 500 $cm^{-1}$): the band at *ca.* 3426 $cm^{-1}$ attributed to ν(OH); the band at *ca.* 2970 $cm^{-1}$ corresponds to ν(C-H); the bands at *ca.* 1619 and 1571 $cm^{-1}$ correspond to asymmetric stretching of carboxylates groups whereas those at *ca.* 1435 and 1378 $cm^{-1}$ correspond to symmetric stretching of the carboxylates groups (Supplementary Fig. S18).[1]

**Transformation of 2 to 1**

Complex **2** (38.96 mg, 0.015 mmol), $Co(NO_3)_2 \cdot 6H_2O$ (34.92 mg, 0.12 mmol), and DMF (6 mL) are mixed in a 20 mL vial at room temperature. The vial is sealed tightly and heated to 50°C, and then the reaction mixture is maintained for 3 days, and cooled down to room temperature. The generation of purple rectangular crystals begins to be observable after 4 hours, and more

formed as the reaction proceeded. Purple rectangular crystals are collected and analyzed through the single crystal X-ray diffraction (SXRD) and powder X-ray diffraction (PXRD) methods.

**Single crystal X-ray diffraction analysis of 1, {[$Co_8(PDA)_6$(*tbu*-PTA)$_3$(DMF)$_4$($H_2O$)$_2$]$_6$-[Co($H_2O$)$_3$]$_4$]}**

The diffraction data from the purple rectangular crystals of **1** (0.180 × 0.140 × 0.090 $mm^3$) mounted on a MiTeGen MicroMount© are collected at 100 K on a ADSC Quantum 210 CCD diffractometer equipped with synchrotron radiation (0.75000 Å) at the Supramolecular Crystallography 2D, Pohang Accelerator Laboratory (PAL), Pohang, Korea. The ADSC Q210 ADX program[2] is used for data collection (detector distance is 63 mm, omega scan; $\Delta\omega = 1°$, exposure time is 1 sec/frame for **1**, and HKL3000sm (Ver. 703r)[3] is used for cell refinement, reduction, and absorption correction. The crystal structure of **1** is solved using the direct method with SHELX-XT (Ver. 2014/5)[4] and refined by full-matrix least-squares calculations with the SHELX-XL (Ver. 2014/7)[5] program package.

The systematic absences in the diffraction data are uniquely consistent for the space group Fd-3 yielding chemically reasonable and computationally stable results of refinement.[5,6]

A successful solution by the direct methods provides most of the non-hydrogen atoms from the *E*-map. The remaining non-hydrogen atoms are located in an alternating series of least-squares cycles and difference Fourier maps. All the non-hydrogen atoms are refined with anisotropic displacement coefficients. All the hydrogen atoms are included in the structure factor calculation at idealized positions and allowed to ride on the neighboring atoms with relative isotropic displacement coefficients. The voids contain disordered DMF with a partial occupancy. A satisfactory disorder model for the solvent is not observed; therefore, the SQUEEZE option of

PLATON[7] is used to mask the disordered density. The squeezed electron density of 7872 $e^-$ per unit cell can be interpreted as 49 DMFs (160 $e^-$). One of the *tert*-butyl groups is observed as disordered and modelled to two different orientations with a partial occupancy of 0.5. The final least-squares refinement of 845 parameters against 20289 data results in residuals $R$ (based on $F^2$ for $I \geq 2\sigma$) and $wR$ (based on $F^2$ for all the data) of 0.0669 and 0.1950, respectively. The final difference Fourier map is featureless.

**Single crystal X-ray diffraction analysis of 2, {$Co_8(PDA)_6$(*tbu*-PTA)$_3$(DMF)$_6$}**

The diffraction data from the purple rhombic crystals of **2** (0.140 × 0.120 × 0.090 $mm^3$) mounted on a MiTeGen MicroMount© are collected at 100 K on a ADSC Quantum 210 CCD diffractometer equipped with synchrotron radiation (0.75000 Å) at the Supramolecular Crystallography 2D, Pohang Accelerator Laboratory (PAL), Pohang, Korea. The ADSC Q210 ADX program[2] is used for data collection (detector distance is 63 mm, omega scan; $\Delta\omega$ = 1°, exposure time is 1 sec/frame for **2**, and HKL3000sm (Ver. 703r)[3] is used for cell refinement, reduction, and absorption correction. The crystal structure of **2** is solved using the direct method with SHELX-XT (Ver. 2014/5)[4] and refined by full-matrix least-squares calculations with the SHELX-XL (Ver. 2014/7)[5] program package.

The systematic absences in the diffraction data are uniquely consistent for the space group $P2_1/n$ yielding chemically reasonable and computationally stable results of refinement.[5,6]

A successful solution by the direct methods provides most of the non-hydrogen atoms from the *E*-map. The remaining non-hydrogen atoms are located in an alternating series of least-squares cycles and difference Fourier maps. All the non-hydrogen atoms are refined with anisotropic displacement coefficients. All the hydrogen atoms are included in the structure factor calculation

at idealized positions and allowed to ride on the neighboring atoms with relative isotropic displacement coefficients. The voids contain disordered DMF with a partial occupancy. A satisfactory disorder model for the solvent is not observed; therefore, the SQUEEZE option of PLATON[7] is used to mask the disordered density. The squeezed electron density of 2906 $e^-$ per unit cell can be interpreted as 18 DMFs (160 $e^-$). One of the coordinated DMF is observed as disordered and modelled to two different orientations with a partial occupancy of 0.5. The final least-squares refinement of 2973 parameters against 59061 data result in residuals $R$ (based on $F^2$ for $I \geq 2\sigma$) and $wR$ (based on $F^2$ for all the data) of 0.0566 and 0.1737, respectively. The final difference Fourier map is featureless.

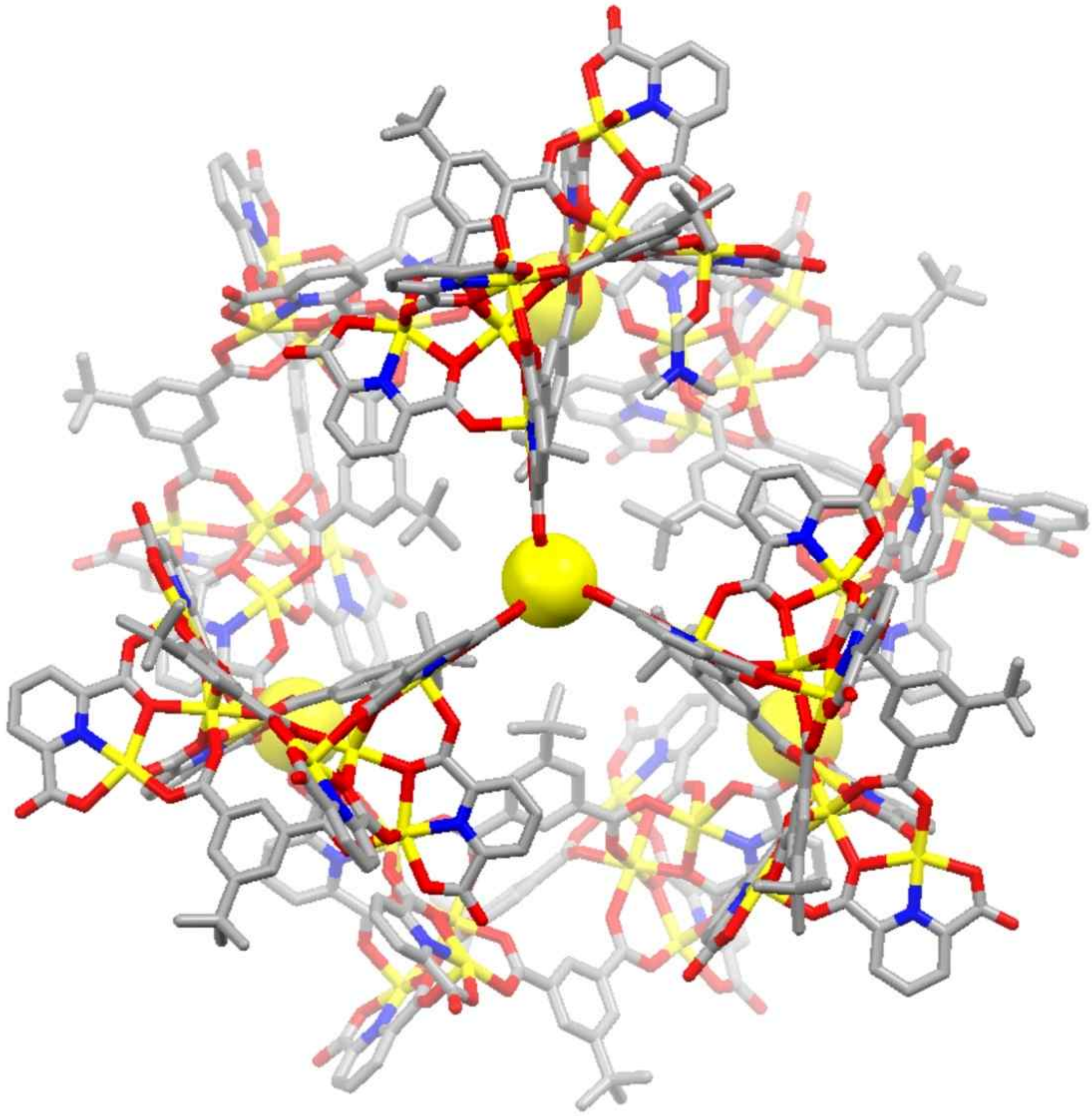

**Fig. S1.** Crystal structure (ball-and-stick diagram) of the discrete molecular cage **1** resulting from the coordination-driven assembly of six Co-TSHs via four Co5 atoms (yellow balls). All hydrogen atoms, coordinated and free solvents, and disordered components on *tbu*-PTA ligands are omitted for clarity. Colour of atoms: C grey, N blue, O red, Co yellow.

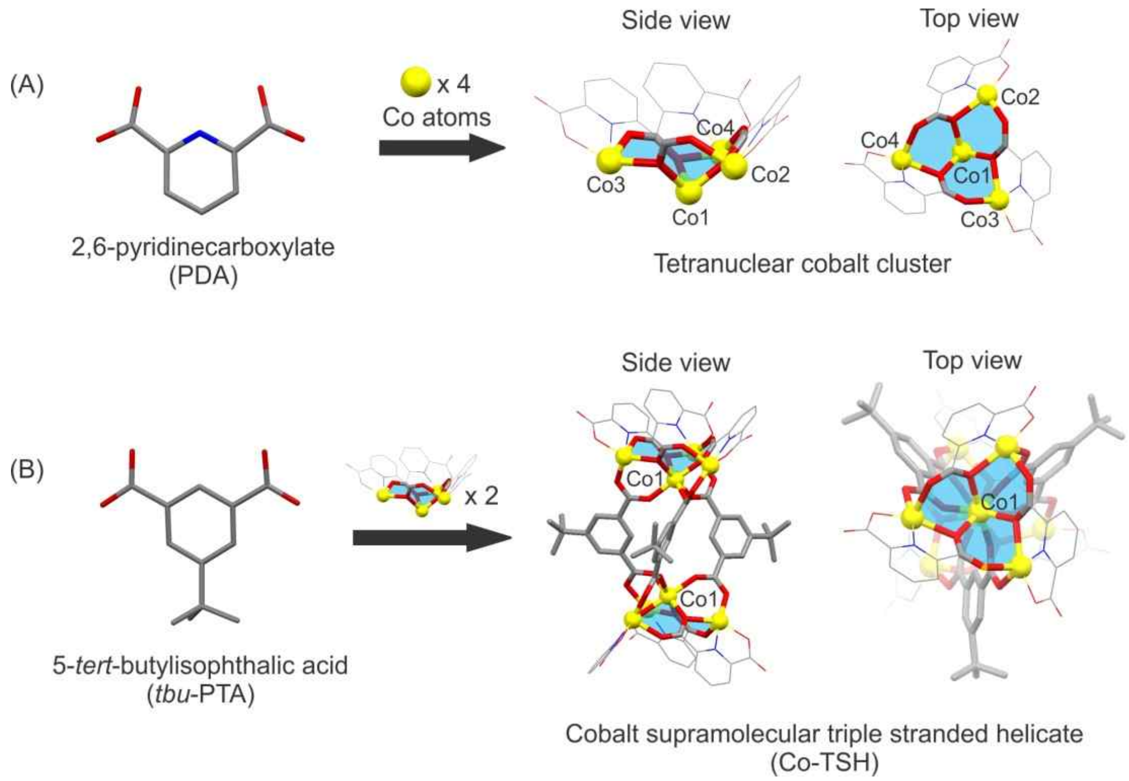


**Fig. S2.** The *in-situ* generation of Co-TSHs acting as supramolecular modules to build the molecular cage **1**. (A) The formation of tetranuclear cobalt clusters from three PDA ligands and four Co atoms. (B) The assembly of two tetranuclear cobalt clusters and three *tbu*-PTA ligands to form a Co-TSH module. All hydrogen atoms, coordinated and free solvents, and disordered components on *tbu*-PTA ligands are omitted for clarity. Colour of atoms: C grey, N blue, O red, Co yellow.

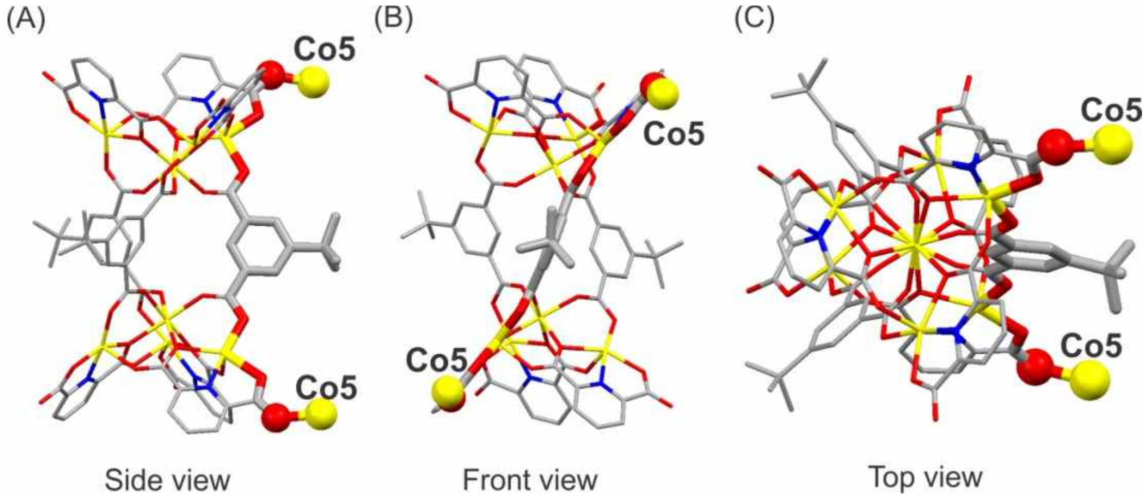


**Fig. S3.** Position and orientation of unoccupied oxygen donors on each Co-TSH module in the molecular cage **1** (crystal structure; ball-and-stick diagram) that directly bound to Co5 atoms. (A) Side, (B) front, and (C) top views of a Co-TSH module. All hydrogen atoms, coordinated and free solvents, and disordered components on *tbu*-PTA ligands are omitted for clarity. Colour of atoms: C grey, N blue, O red, Co yellow.

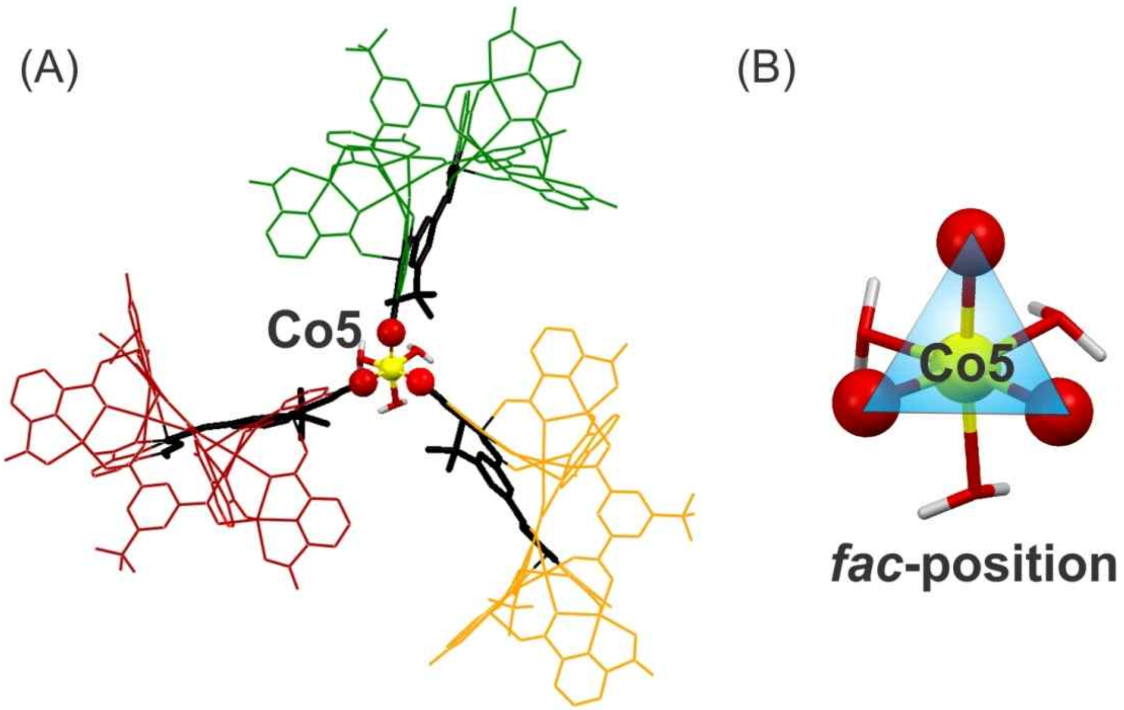


**Fig. S4.** The coordination environment of linking Co5 atoms. (A) Each Co5 atom interconnects its three neighboring Co-TSHs that are coded in different colors. (B) Each Co5 atom adopting octahedral coordination geometry bounds to three carboxylate oxygen atoms (red balls) from three PDA ligands of three neighboring Co-TSHs in a facial *(fac-)* mode. Colour of atoms: H white, O red, Co yellow.

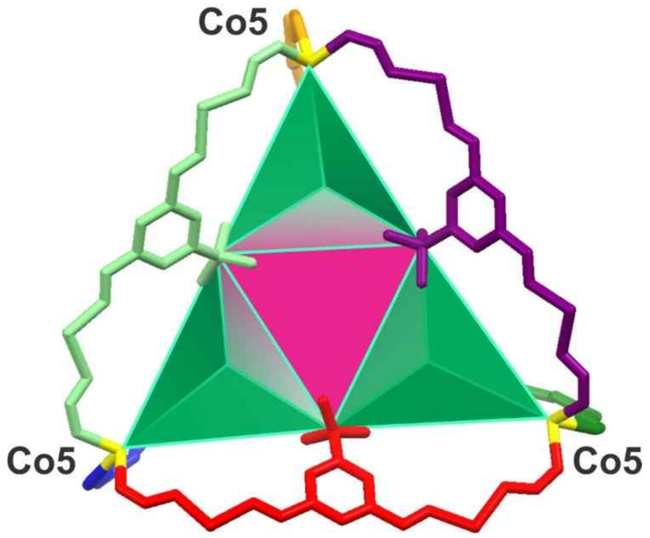


**Fig. S5.** Schematic representation showing the large tetrahedral space of the molecular cage **1** composed of two types of smaller voids: one octahedron and four tetrahedrons (three in the foreground and one in the background). Octahedral void and tetrahedrons are coloured pink and green, respectively.

The tetrahedral space of the molecular cage **1** comprises two types of smaller voids (one octahedron and four tetrahedrons assigned as *O*- and *T*-voids, respectively). The former, defined by six quaternary carbon atoms (each belonging to a *tert*-butyl group of a Co-TSH that directs to the centre), is arranged at the centroid of **1** with an average edge distance of *ca.* 8 Å. Moreover, four *T*-voids, each is enclosed by one Co5 atom and three quaternary carbon atoms from the *tert*-butyl groups of three neighboring Co-TSHs, has an average edge distance of *ca.* 8.1 Å. These *O*- and *T*-voids are face-sharing.

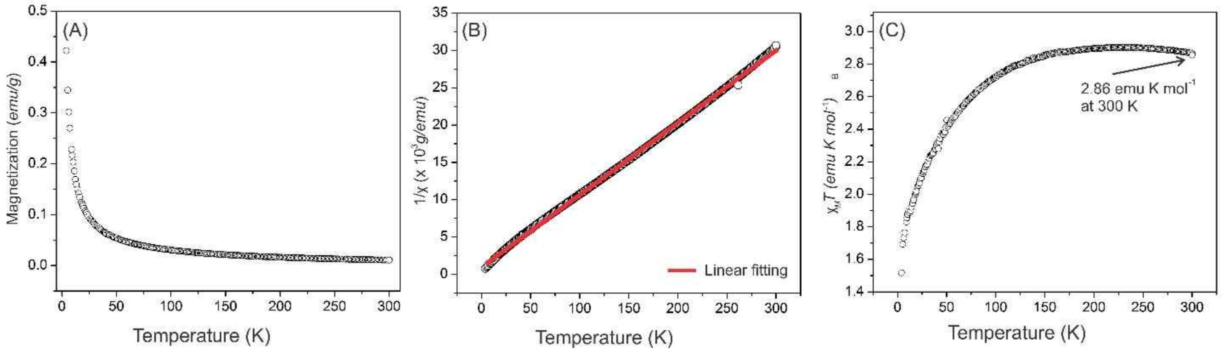


**Fig. S6.** (A) Temperature-dependent magnetization, (B) Curie-Weiss fitting (red line) of the inverse mass susceptibility ($1/\chi$) versus temperature, and (C) plot of $\chi_M T$ versus $T$ of complex **1**.

The magnetic behavior of complex **1** could be described by Curie-Weiss law:[8-11]

$$\chi = \frac{C}{T-\theta} \qquad \text{(eq. 1)}$$

where $\chi$ is mass susceptibility (emu $g^{-1}$); $C$ is the Curie-Weiss constant (emu K $g^{-1}$); $T$ is temperature (K); $\theta$ is the Weiss constant (K). The mass susceptibility of the compound is related to the molar susceptibility (emu $mol^{-1}$), $\chi_M$:

$$\chi_M = \chi \,.\, \mathrm{MW}/Z \qquad \text{(eq.2)}$$

where MW is the molecular weight of the compound and $\boldsymbol{Z}$ is the number of moles of magnetic ions per formula weight of the compound (for the calculation, the chemical formula for **1**, $C_{540}H_{540}Co_{52}N_{60}O_{264}$, is used).

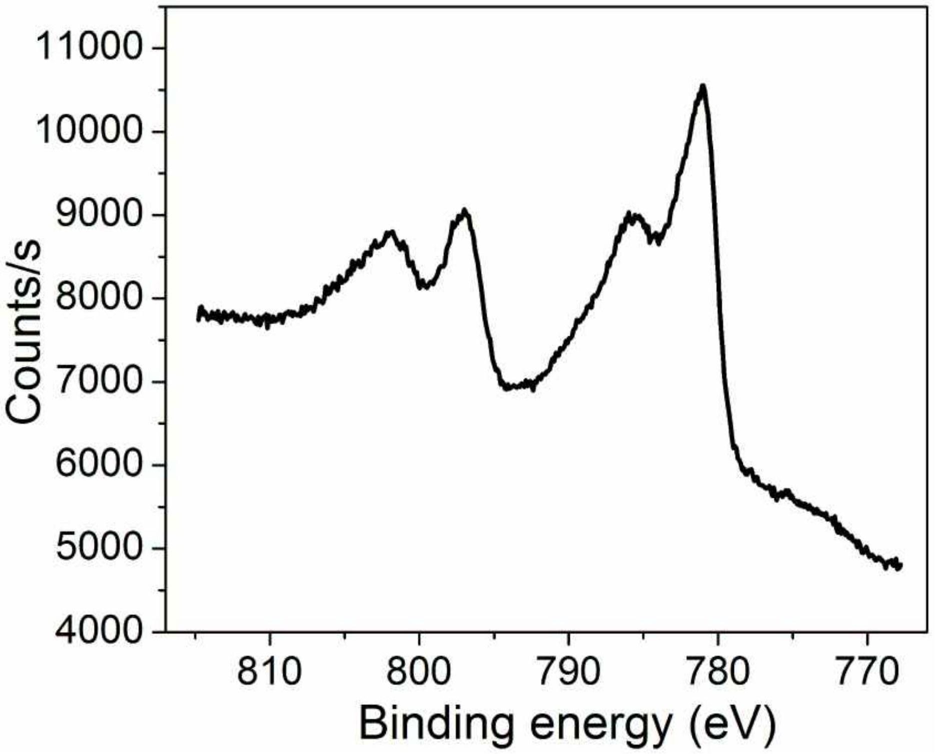


**Fig. S7.** Co 2p XPS data of complex **1**.

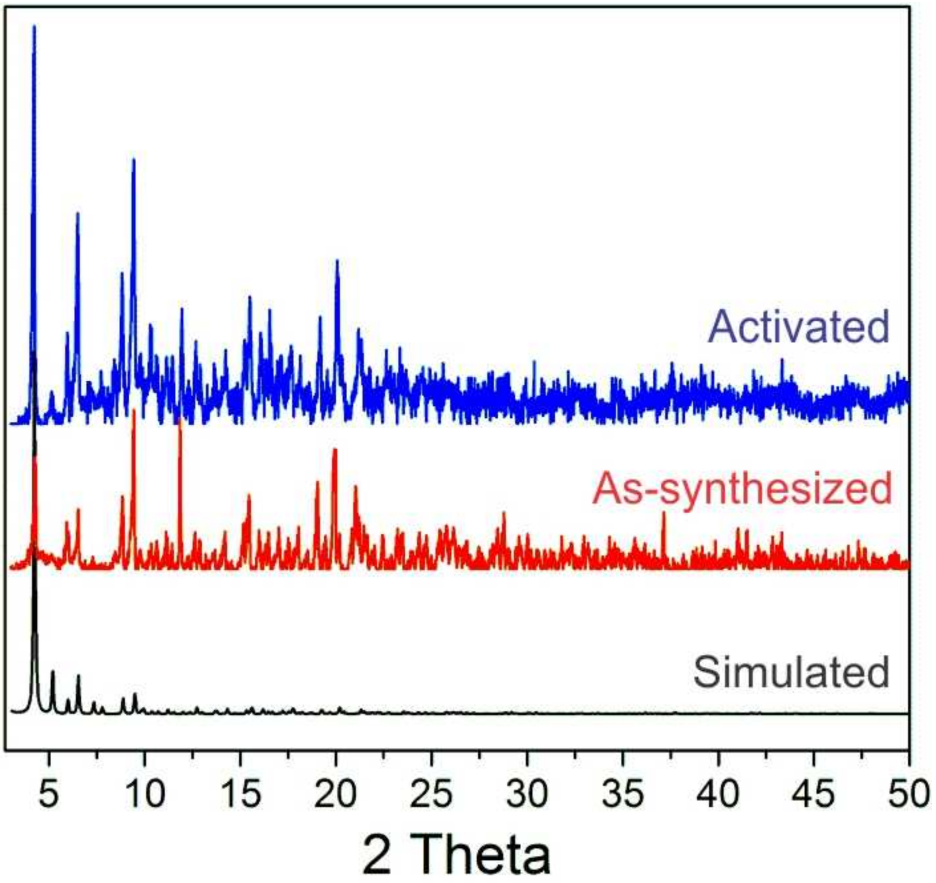


**Fig. S8.** PXRD patterns for the molecular cage **1**. Pattern simulated from single-crystal structure is shown in black; experimental patterns for as-synthesized in red and activated (heating at 60 °C under a vacuum for 24 hours) in blue, respectively.

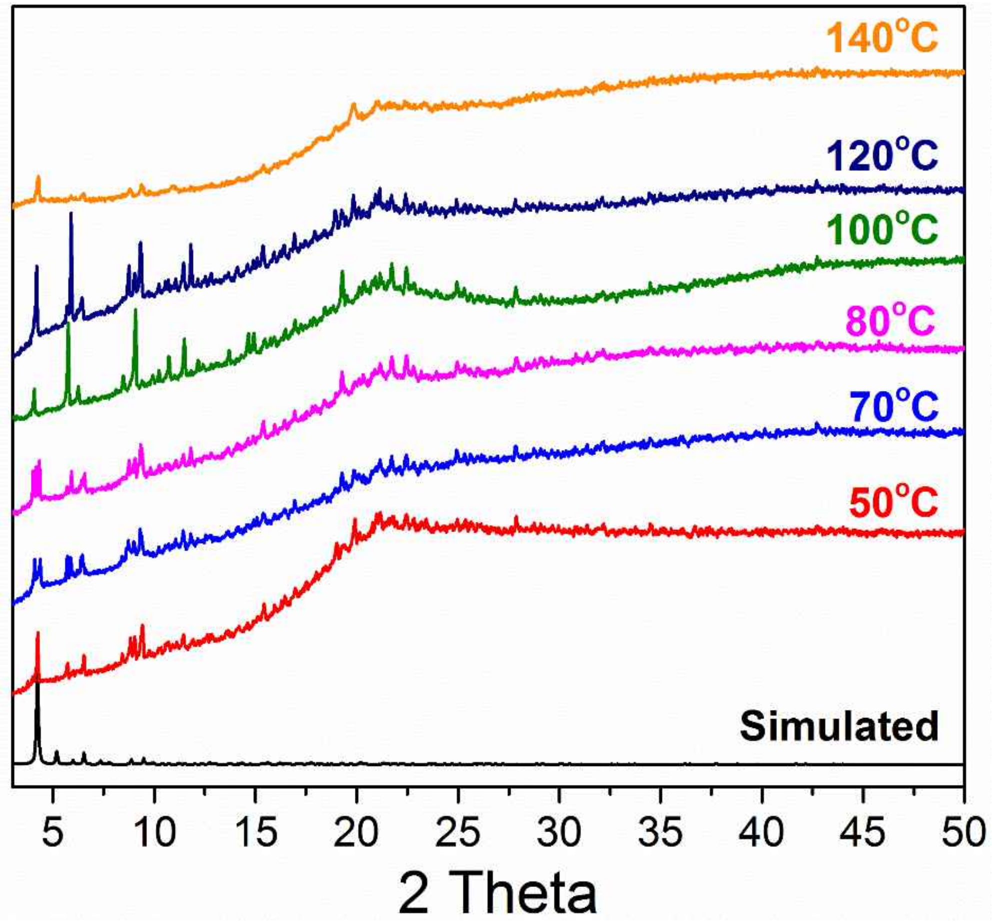


**Fig. S9.** PXRD patterns for the molecular cage **1** at different treating temperatures (50, 70, 80, 100, 120, and 140 °C) in DMF. Pattern simulated from single-crystal structure is shown in black.

- As-prepared samples are thoroughly washed with 5 mL of DMF (1 time) and redispersed in 12 mL of DMF. This mixture is subjected to thermal treatment at different temperatures (50, 70, 80, 100, 120, and 140 °C) for 24 hours. PXRD experiments are done using wet samples (with DMF).

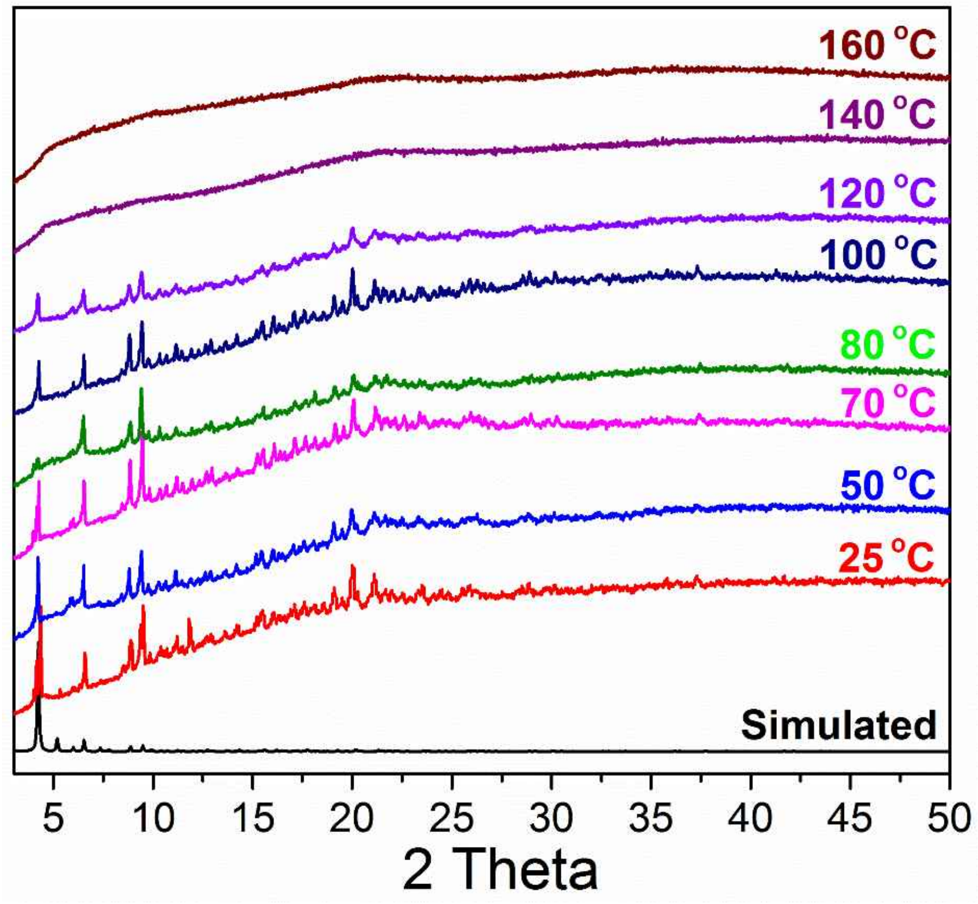


**Fig. S10.** PXRD patterns for the molecular cage **1** at different temperatures (25, 50, 70, 80, 100, 120, 140, and 160 °C) for 8 hours in the air. Pattern simulated from single-crystal structure is shown in black.

- As-prepared samples are washed thoroughly with 5 mL of DMF (3 times) and 5 mL of acetone (3 times). Activation of washed samples is carried out at different temperatures (25, 50, 70, 80, 100, 120, 140, and 160 °C) for 8 hours in the air. PXRD experiments are done using dried samples.

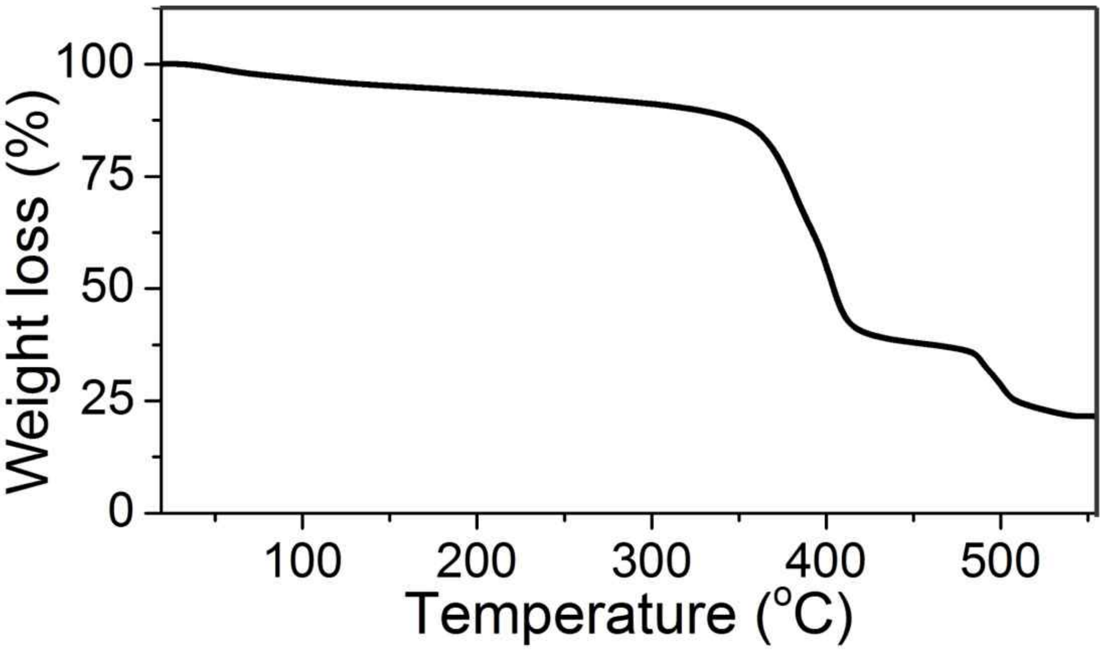


**Fig. S11.** Thermogravimetric analysis (TGA) for the molecular cage **1**.

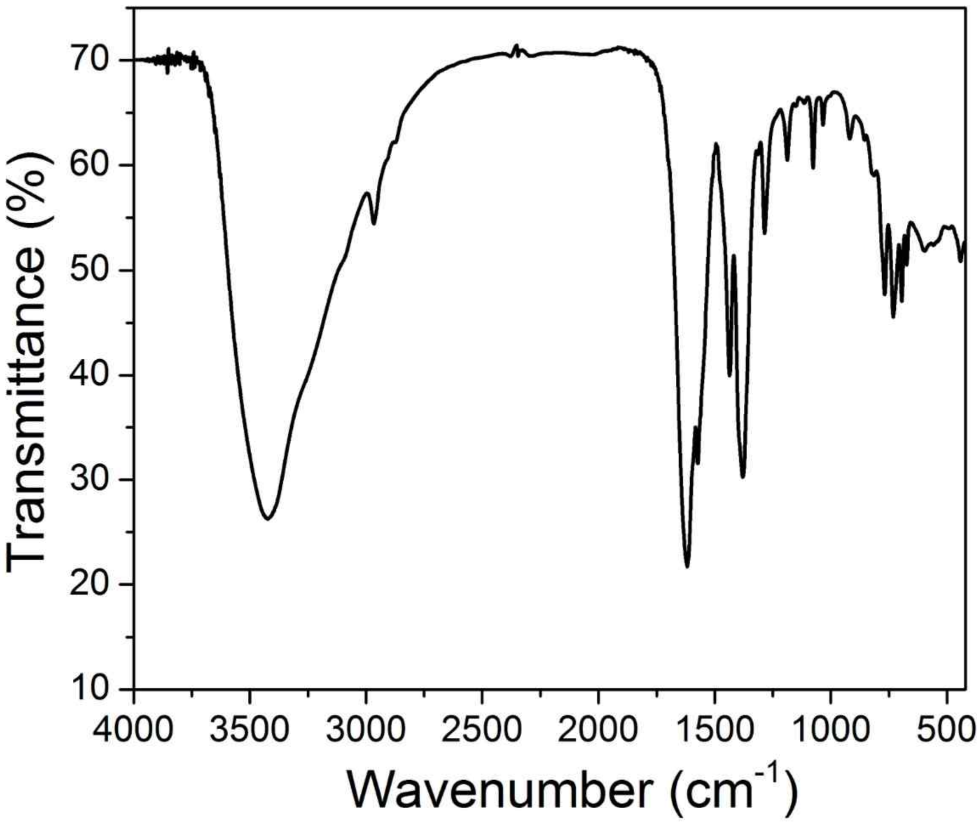


**Fig. S12.** FTIR spectrum of the molecular cage **1**.

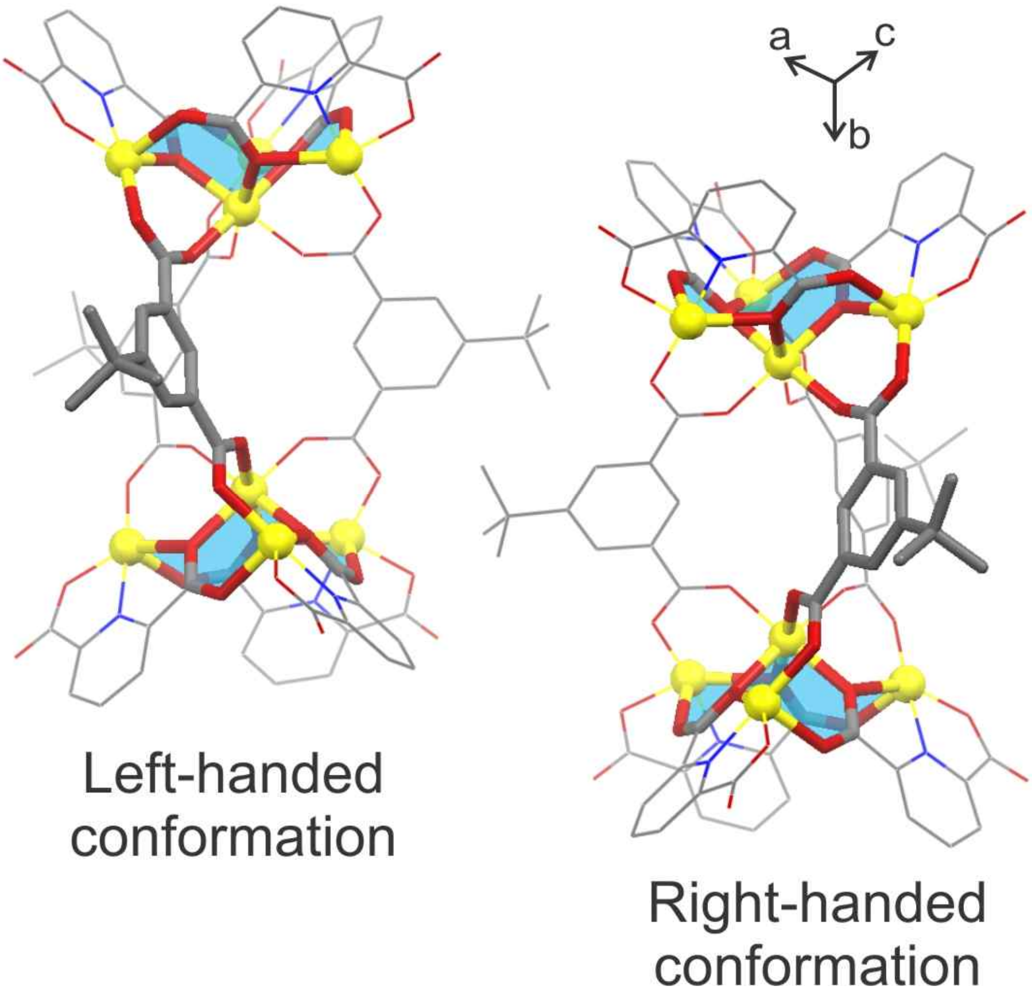


**Fig. S13**. Crystal structure (ball-and-stick diagram) of complex **2** showing two distinct conformations existing simultaneously in a single unit cell. All hydrogen atoms, coordinated and free solvents, and disordered components on a coordinated DMF molecule are omitted for clarity. Colour of atoms: C grey, N blue, O red, Co yellow.

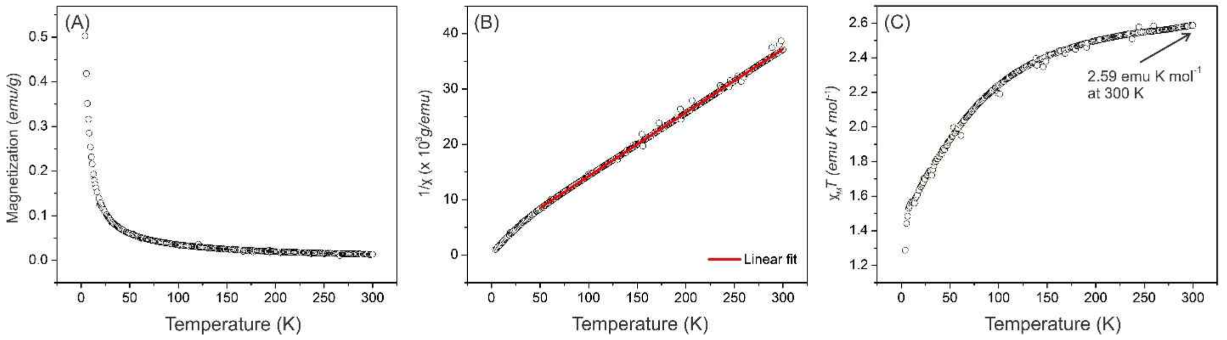


**Fig. S14.** (A) Temperature-dependent magnetization, (B) Curie-Weiss fitting (red line) of the inverse mass susceptibility ($1/\chi$) versus temperature, and (C) plot of $\chi_M T$ versus $T$ of complex **2**.

Calculation of the molar susceptibility ($\boldsymbol{\chi_M}$) of the compound **2** is similar to that for compound **1**, except that the chemical formula for **2**, $C_{96}H_{96}Co_8N_{12}O_{42}$, is used.

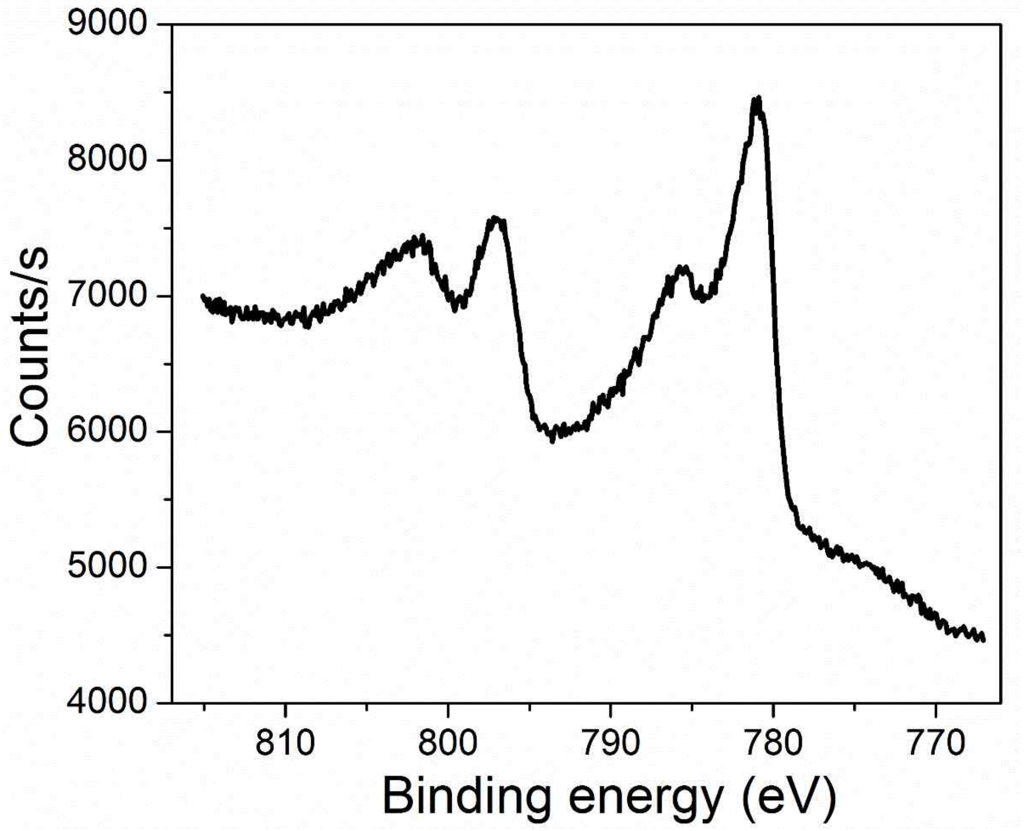


**Fig. S15.** Co 2p XPS data of complex **2**.

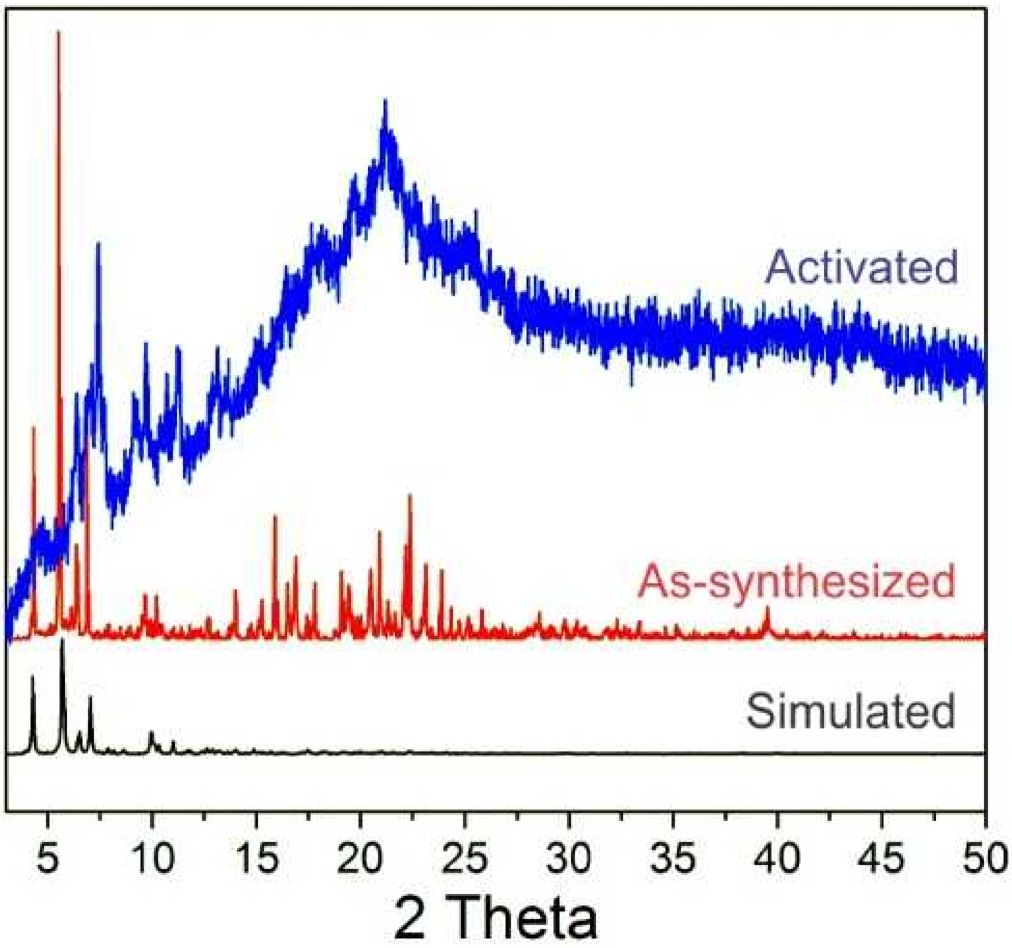


**Fig. S16.** PXRD patterns for complex **2**. Pattern simulated from single-crystal structure is shown in black; experimental patterns for as-synthesized in red and activated (heating at 60 °C under a vacuum for 24 hours) in blue, respectively.

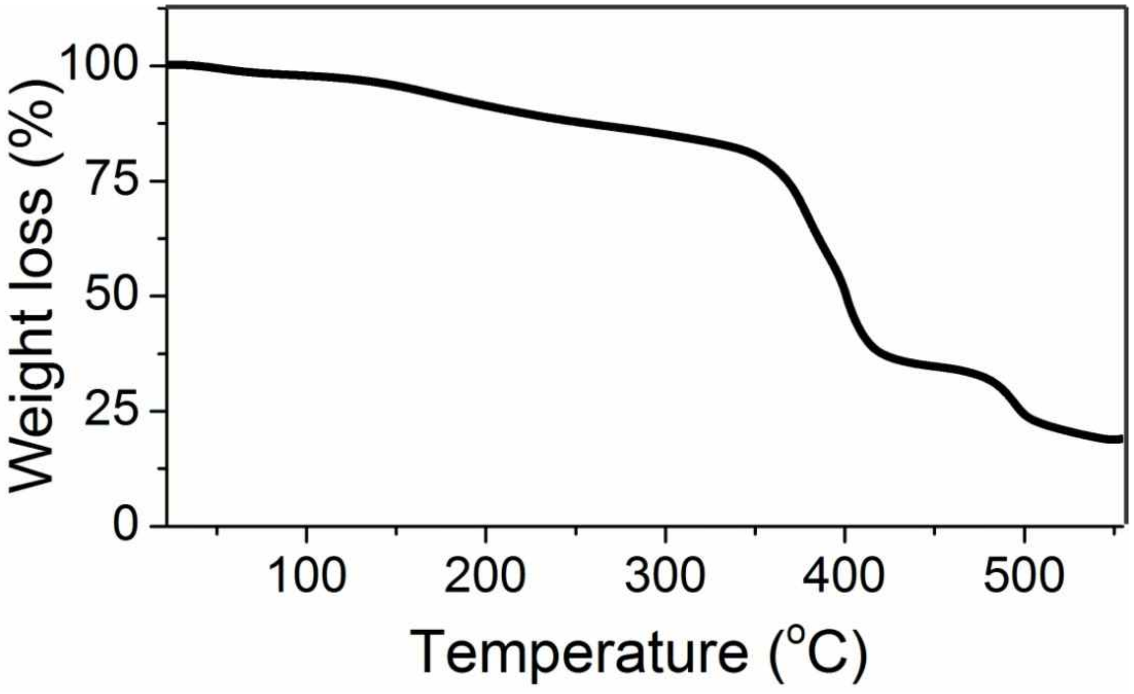


**Fig. S17.** Thermogravimetric analysis (TGA) for complex **2**.

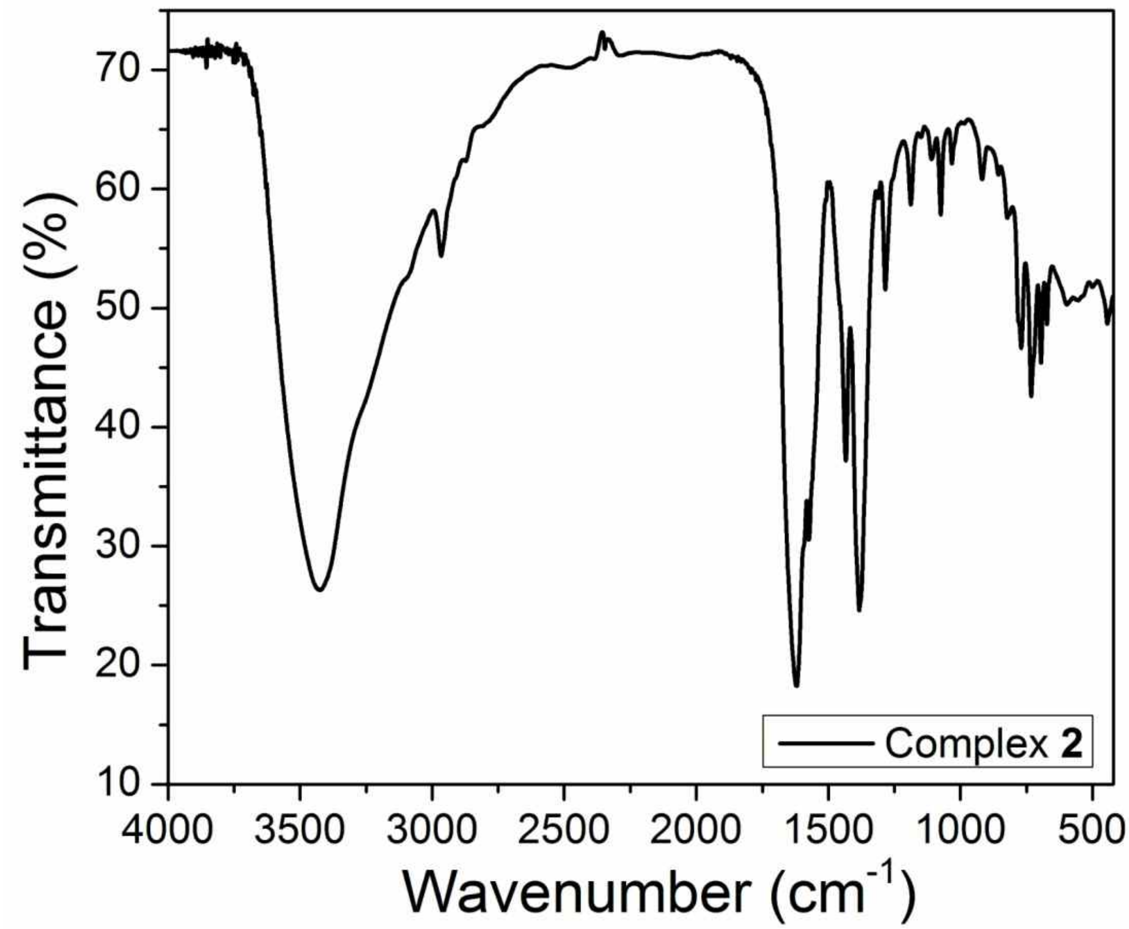


**Fig. S18.** FTIR spectrum of complex **2**.

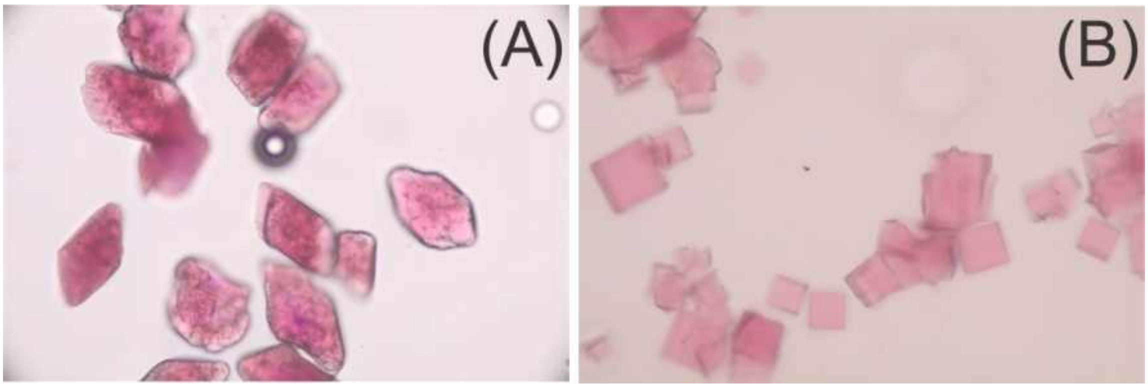


**Fig. S19.** Microscopic images of crystals pictured (A) before and (B) after treatment of complex **2** with $Co(NO_3)_2{\cdot}6H_2O$ in DMF at 50 °C for 3 days. Formation of rectangular crystals (in B) indicates the transformation of complex **2** to the molecular cage **1**.

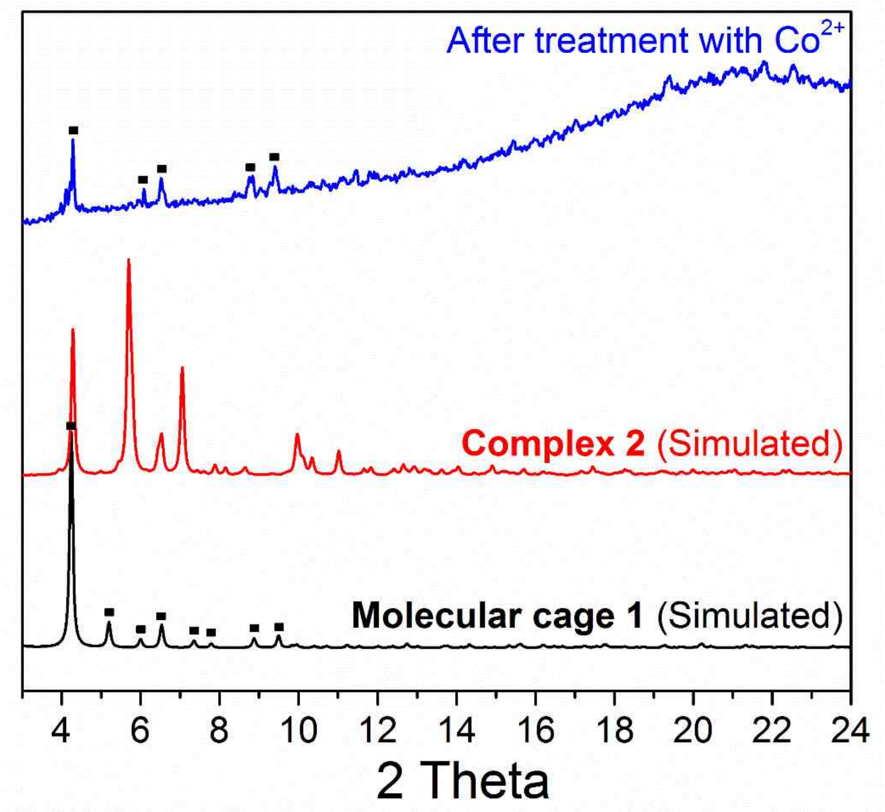


**Fig. S20.** Experimental PXRD patterns (in blue) for the crystals collected after treatment of complex **2** with $Co(NO_3)_2 \cdot 6H_2O$ in DMF at 50 °C for 3 days. Patterns simulated from single-crystal structures of **1** and **2** are shown in black and red, respectively.

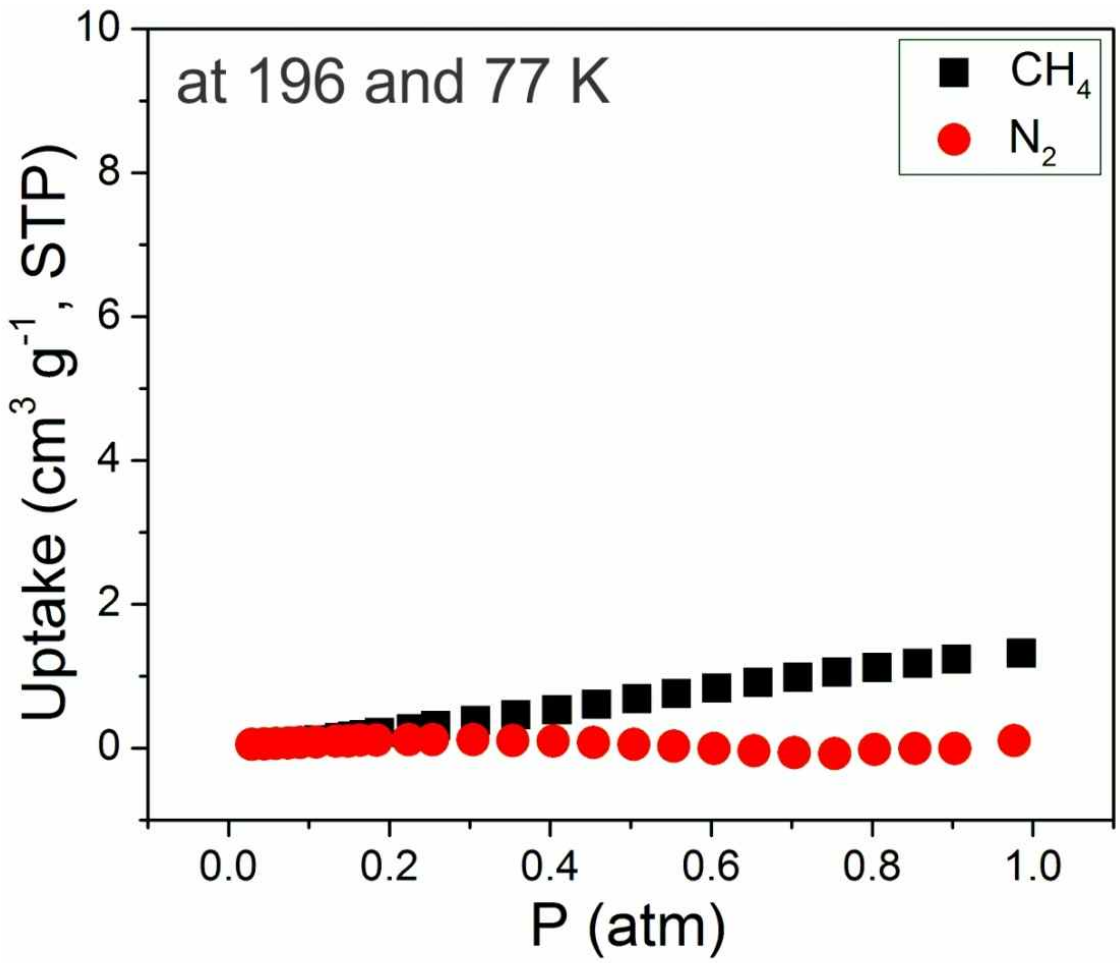


**Fig. S21.** Adsorption isotherms for $CH_4$, and $N_2$ of complex **2** collected at 196 K and 77 K, respectively.

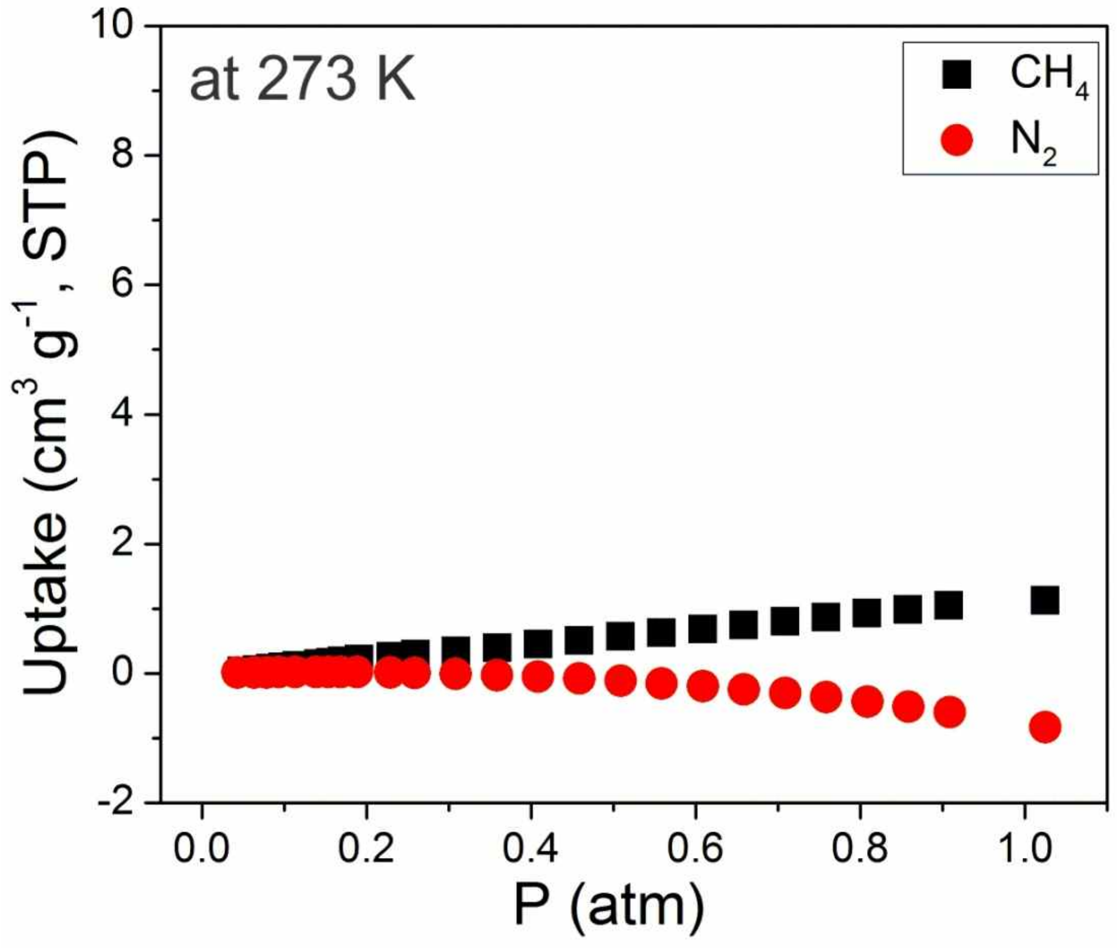


**Fig. S22.** Adsorption isotherms for $CH_4$, and $N_2$ of complex **2** collected at 273 K.

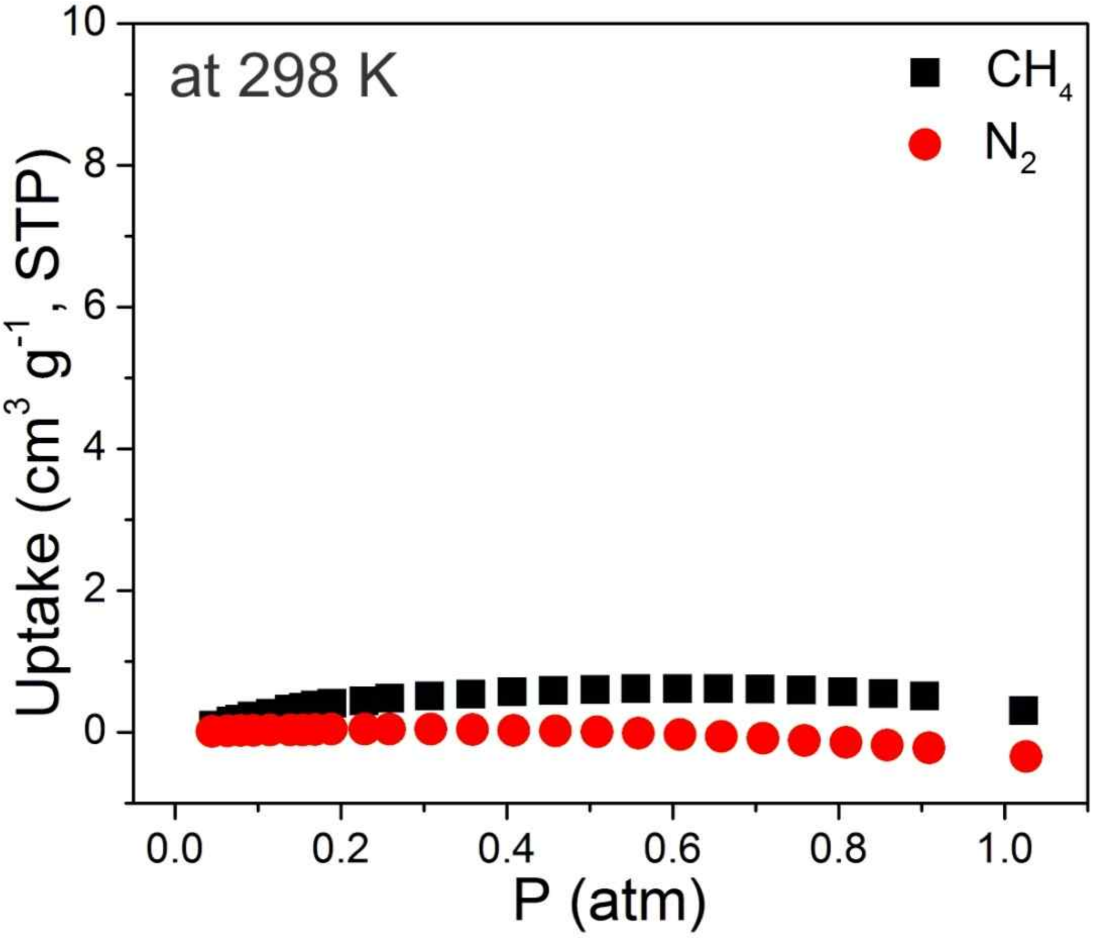


**Fig. S23.** Adsorption isotherms for $CH_4$, and $N_2$ of complex **2** collected at 298 K.

**Table S1**. Crystal data and structure refinement for the molecular cage **1**; atomic coordinates, equivalent isotropic displacement parameters, anisotropic displacement parameters, bond lengths, and angles shown in the CIF.

| | |
|---|---|
| Empirical formula | $C_{540}H_{540}Co_{52}N_{60}O_{264}$, 24($C_3H_7NO$) |
| Formula weight | 16912.94 |
| Temperature/K | 100(2) |
| Crystal system | cubic |
| Space group | Fd-3 |
| a/Å | 58.880(7) |
| b/Å | 58.880(7) |
| c/Å | 58.880(7) |
| α/° | 90 |
| β/° | 90 |
| γ/° | 90 |
| Volume/Å$^3$ | 204133(71) |
| Z | 8 |
| $\rho_{calc}$g/cm$^3$ | 1.101 |
| μ/mm$^{-1}$ | 1.025 |
| F(000) | 69408.0 |
| Crystal size/mm$^3$ | 0.180 × 0.140 × 0.090 |
| Radiation | synchrotron (λ = 0.75000) |
| 2Θ range for data collection/° | 4.842 to 59.292 |
| Index ranges | $-77 \leq h \leq 77$, $-53 \leq k \leq 77$, $-77 \leq l \leq 54$ |
| Reflections collected | 217958 |
| Independent reflections | 20289 [$R_{int}$ = 0.0553, $R_{sigma}$ = 0.0215] |
| Data/restraints/parameters | 20289/75/845 |
| Goodness-of-fit on F$^2$ | 1.041 |
| Final R indexes [I>=2σ (I)] | $R_1$ = 0.0669, $wR_2$ = 0.1936 |
| Final R indexes [all data] | $R_1$ = 0.0692, $wR_2$ = 0.1950 |
| Largest diff. peak/hole / e Å$^{-3}$ | 1.61/-0.77 |

**Table S2**. Crystal data and structure refinement for complex **2**; atomic coordinates, equivalent isotropic displacement parameters, anisotropic displacement parameters, bond lengths, and angles shown in the CIF.

| | |
|---|---|
| Empirical formula | $2(C_{96}H_{96}Co_8N_{12}O_{42})$, $C_3H_7NO$ |
| Formula weight | 5195.66 |
| Temperature/K | 100(2) |
| Crystal system | monoclinic |
| Space group | $P2_1/n$ |
| a/Å | 30.348(6) |
| b/Å | 30.990(6) |
| c/Å | 35.655(7) |
| α/° | 90 |
| β/° | 114.55(3) |
| γ/° | 90 |
| Volume/Å$^3$ | 30503(12) |
| Z | 4 |
| $\rho_{calc}$g/cm$^3$ | 1.131 |
| μ/mm$^{-1}$ | 0.915 |
| F(000) | 10624.0 |
| Crystal size/mm$^3$ | 0.140 × 0.120 × 0.090 |
| Radiation | synchrotron (λ = 0.75000) |
| 2Θ range for data collection/° | 4.786 to 56 |
| Index ranges | $0 \leq h \leq 39$, $-40 \leq k \leq 40$, $-46 \leq l \leq 39$ |
| Reflections collected | 133098 |
| Independent reflections | 59061 [$R_{int}$ = 0.0363, $R_{sigma}$ = 0.0390] |
| Data/restraints/parameters | 59061/56/2972 |
| Goodness-of-fit on F$^2$ | 1.083 |
| Final R indexes [I>=2σ (I)] | $R_1$ = 0.0566, $wR_2$ = 0.1686 |
| Final R indexes [all data] | $R_1$ = 0.0633, $wR_2$ = 0.1737 |
| Largest diff. peak/hole / e Å$^{-3}$ | 1.29/-1.11 |

**Table S3.** The calculated values through the bond valence sum analysis for cobalt ions in complex **1**.[12,13]

| Cobalt atoms in Complex **1** | Bond valence sum |
|---|---|
| Co1 | 2.01 |
| Co2 | 2.00 |
| Co3 | 2.06 |
| Co4 | 2.10 |
| Co5 | 1.95 |

**Table S4.** The calculated values through the bond valence sum analysis for cobalt ions in left- and right-handed conformations of complex **2**.[12,13]

| Cobalt atoms in left-handed conformation | Bond valence sum | Cobalt atoms in right-handed conformation | Bond valence sum |
|---|---|---|---|
| Co1 | 1.97 | Co9 | 2.00 |
| Co2 | 2.07 | Co10 | 2.04 |
| Co3 | 2.05 | Co11 | 2.05 |
| Co4 | 2.06 | Co12 | 2.03 |
| Co5 | 1.99 | Co13 | 1.99 |
| Co6 | 2.06 | Co14 | 2.06 |
| Co7 | 2.06 | Co15 | 2.04 |
| Co8 | 2.07 | Co16 | 2.10 |

**Table S5.** $CO_2$ uptakes of the molecular cage **1** and other selected discrete molecular platforms constructed from metal ions/clusters and multifunctional organic ligands at close room temperature and 1 atm.

| **Compound** | **Adsorption Temperature (K)** | **$CO_2$ Uptake at 1 atm ($cm^3\ g^{-1}$)** | **Ref** |
|---|---|---|---|
| Molecular cage **1** | 273 | 83 | *This work* |
| | 298 | 61 | |
| $[Co_{24}(BTC4A)_6(\mu_4\text{-}Cl_6)(BDC)_{12}]^{6-}$ | 298 | 25 | (14) |
| $\{[(Ni_4(\mu_4\text{-}H_2O)(DTBSC)]_6(BTC)_8\}$ | 293 | 30 | (15) |
| $[Et_3NH]_2\{[Co_4(TC4A)(Cl)]_2[M_4(TC4A)(SO_4)]_4bpdc_8\}$ | 273 | 80 | (16) |
| $(NH_2Me_2)_6[(V_4O_8Cl)_6(BTC)_8]$ | 298 | 27 | (17) |
| $[Na_2(BTC4A)_6(BIPY)_3(CO_3)_6(OH)_8(Cl)_4(H_2O)_{10}(dma)_8].10\ OAc$ | 273 | 53.36 | (18) |
| $[(CH_3)_4N]_3\{(Ni_4\text{-}TC4A)_{10}(Cl)_{16}(PIP)_{16}(HPIP)_{0.775}(HCOO)_{1.225}(CO_3)_{0.9868}(MeNCOO)_{1.0132}(DMF)_{0.93924}\}$ | 273 | 59.7 | (19) |
| | 298 | 32.7 | |
| $Cu_{24}(2,7\text{-}NDC)_{24}(DEF)_8(H_2O)_{16}\cdot(DEF)_{20}(EtOH)_4(H_2O)_{14}$ | 273 | 50 | (20) |
| $[Cu_2(2,7\text{-}NDDB)_2(DMA)_2]_2$ | 273 | 19 | (21) |
| | 298 | 14 | |
| $[Cu(5\text{-}TE\text{-}BDC)(CH_3OH)(H_2O)]_{24}$ | 273 | 25 | (22) |
| $[Cu_{24}(L)_{24}(H_2O)_{16}(DMA)_8]$ | 298 | 7 | (23) |

**Reference**


(1) Fei, H.; Liu, X.; Li, Z.; Feng, W. *Dalton Trans.* **2015**, *44*, 9909.
(2) Arvai, A. J. & Nielsen, C. "ADSC Quantum-210 ADX Program" (Area Detector System Corporation: Poway, CA, USA, 1983).
(3) Otwinowski, Z. & Minor, W. Processing of X-ray diffraction data collected in oscillation mode. Methods in Enzymology, Volume 276: Macromolecular Crystallography, part A, p.307-326, 1997, C.W. Carter, Jr. & R. M. Sweet, Eds., Academic Press (New York).
(4) Sheldrick, G. M. SHELXT - integrated space-group and crystal structure. *Acta Cryst. A* **2015**, *71*, 3.
(5) Sheldrick, G. M. Crystal structure refinement with SHELXL. *Acta. Cryst. C* **2015**, *71*, 3.
(6) Dolomanov, O. V.; Bourhis, L. J.; Gildea, R. J.; Howard, J. A. K.; Puschmann, H. J. OLEX2: a complete structure solution, refinement and analysis program. *Appl. Cryst.* **2009**, *42*, 339.
(7) Spek, A. L. PLATON SQUEEZE: a tool for the calculation of the disordered solvent contribution to the calculated structure factors. *Acta Cryst. C* **2015**, *71*, 9.
(8) Carlin, R. L. *Magnetochemistry*, Springer-Verlag, Berlin, 1986.
(9) Selwood, P. W. *Magnetochemistry*; Interscience: New York, 1956.
(10) Shen, X.-F., Ding, Y.-S., Liu, J., Han, Z.-H., Budnick, J. I., Hines, W. A., Suib. S. L. *J. Am. Chem. Soc.* 2005, 127, 6166.
(11) Teweldemedhin, Z. S., Fuller, R. L., Greenblatt, M. *J. Chem. Educ.* 1996, 73, 906.
(12) Palenik, G. J. *Inorg. Chem.* **1997**, *36*, 122.
(13) O'Keefe, M., Brese, N. E. *J. Am. Chem. Soc.* **1991**, *113*, 3226.
(14) Xiong, K.; Jiang, F.; Gai, Y.; Yuan, D.; Chen, L.; Wu, M.; Su K.; Hong, M. Chem. Sci. 2012, 3, 2321.
(15) Dai, F.-R.; Wang, Z. *J. Am. Chem. Soc.* **2012**, *134*, 8002.
(16) Tan, H.; Du, S.; Bi, Y.; Liao, W. *Inorg. Chem.* **2014**, *53*, 7083.
(17) Zhang, Z.; Wojtas, L.; Zaworotko, M. J. *Chem. Sci.* **2014**, *5*, 927.
(18) Su, K.; Jiang, F.; Qian, J.; Chen, L.; Pang, J.; Bawaked, S. M.; Mokhtar, M.; Al-Thabaiti, S. A.; Hong, M. *Inorg. Chem.* **2015**, *54*, 3183.
(19) Hang, X.; Liu, B.; Zhu, X.; Wang, S.; Han, H.; Liao, W.; Liu, Y.; Hu, C. *J. Am. Chem. Soc.* **2016**, *138*, 2969.
(20) Furukawa, H.; Kim, J.; Ockwig, N. W.; O'Keeffe, M.; Yaghi, O. M. *J. Am. Chem. Soc.* **2008**, *130*, 11650.
(21) Li, J.-R.; Yu, J.; Lu, W.; Sun, L.-B.; Sculley, J.; Balbuena, P. B.; Zhou, H.-C. *Nat. Commun.* **2013,** DOI: 10.1038/ncomms2552.
(22) Zhao, D.; Yuan, D.; Krishna, R.; Baten J. M. V.; Zhou, H.-C. *Chem. Commun.* **2010**, *46*, 7352.
(23) Niu, Z.; Fang, S.; Liu, X.; Ma, J.-G.; Ma S.; Cheng, P. *J. Am. Chem. Soc.* **2015**, *137*, 14873.
